# Modelling of High Velocity Impact on Concrete Structures Using a Rate-Dependent Plastic-Damage Microplane Approach at Finite Strains

**DOI:** 10.3390/ma13225165

**Published:** 2020-11-16

**Authors:** Bobby Rio Indriyantho, Imadeddin Zreid, Robert Fleischhauer, Michael Kaliske

**Affiliations:** Institute for Structural Analysis, Technische Universität Dresden, 01062 Dresden, Germany; bobby_rio.indriyantho@tu-dresden.de (B.R.I.); imadeddin.zreid@tu-dresden.de (I.Z.); robert.fleischhauer@tu-dresden.de (R.F.)

**Keywords:** concrete, microplane, plastic-damage model, finite strain, implicit gradient enhancement, rate dependency

## Abstract

Concrete is known as a quasi-brittle material and the microplane model has been proven to be a powerful method to describe its constitutive features. For some dynamic cases, however, numerous microplane models used successfully at small strains are not sufficient to predict the nonlinear behaviour of damaged concrete due to large deformations. In this contribution at hand, a combined plasticity-damage microplane model extended to the finite strain framework is formulated and regularised using implicit gradient enhancement to achieve mesh insensitivity and to obtain more stable finite element solutions. A modified smooth three surface Drucker–Prager yield function with caps is introduced within the compression-tension split. Moreover, a viscoplastic consistency formulation is implemented to deliver rate dependency at dynamic cases. In case of penetration into concrete materials, the proposed model is equipped with an element erosion procedure to yield a better approximation of crack patterns. Numerical examples on impact cases are performed to challenge the capability of the newly proposed model to existing experimental data.

## 1. Introduction

Throughout a building’s service period, complex behaviour can be observed in concrete structures, such as for instance military base anchorages, nuclear reactors, long-span bridges, and oil platforms, which are subjected to impact phenomena, natural disasters such as earthquake or seismic loading, detonation, or blast loading with high intensity. These circumstances lead to dynamic fractures in concrete material up to the final failure of its structure. Finite element simulations are capable of modelling various phenomena in concrete using material modelling developed extensively up to now. Concrete itself is known as a quasi-brittle material with heterogeneous complex nature as a result of its constituent materials, such as cement paste and different type of aggregates. The behaviour of concrete changes from linear elastic to highly nonlinear inelastic due to several physical phenomena as microcracking, crushing or even plasticity caused by tension, compression, or combination of both tension and compression. These conditions yield difficulties in modelling concrete, especially in terms of constitutive laws and numerical aspects regarding the finite element method.

Several approaches for modelling these phenomena have been employed in order to describe concrete responses realistically in the context of continuum mechanics. The most renowned approaches are using continuum damage mechanics, plasticity, or a combination of damage and plasticity. Continuum damage mechanics is characterised by the degradation of the material stiffness describing the inelastic behaviour at post-peak range. Meanwhile, plasticity deals with elastic-plastic conditions as a result of the stress state, which are evaluated with respect to the applied yield criterion. At monotonic loadings, in fact, either damage or plasticity models are sufficient to represent softening behaviour of concrete. Nonetheless, by the fact that a structure is subjected not only to monotonic loading but also to unloading-reloading states altogether, the necessity of a coupled plastic-damage formulation is inevitable. Various plastic-damage approaches for concrete can be found in [1,2,3,4,5,6,7,8,9]. Moreover, other plastic-damage models with different features are also available, e.g. for impact failure [10], for concrete spalling considering creep-shrinkage [11], for ductile failure [12], and for granular materials at low confining pressure [13].

In the last three decades, a powerful model for concrete materials, namely the microplane approach, has been studied and developed extensively since it was pioneered by [14]. The concept of microplanes is characterised by a projection of the strain tensor to random planes of a microscale. This concept is straightforward to understand the relation between strain and stress vectors in each microplane, which is eventually equivalent to macroscopic strains and stresses. This approach was then improved by [15], namely the model M1 and subsequently, it was developed further up to the recent version M7 employing stress-strain bounds [16]. As discussed in [17], however, the conventional microplane models using either the normal-tangential (N-T) or the volumetric-deviatoric-tangential (V-D-T) split have several mechanical deficits. The full range of Poisson’s ratio cannot be represented by the N-T split, while microplane elastic constants with the unique macroscopic-microplane relation is not possible to be considered by the V-D-T split. As observed in [18], the ambiguity of the deviatoric-tangential projection allows a lot of freedom in the V-D-T split and it leads to problems in numerical implementation. Therefore, the deviatoric and tangential components in the V-D-T split were merged and resulted in the volumetric-deviatoric (V-D) split, see [19].

For strain softening at the post-peak regime, however, problems relating to ill-posed differential equations and strain localisation occur. In consequence, these issues lead to pathological mesh dependencies and unstable finite element solutions eventually indicated by slow convergence rates. The so-called implicit gradient enhancement introduced in [20,21] has been proven to tackle these problems, and it has been successfully implemented to regularise the microplane damage model using the V-D split in [22]. Moreover, the gradient-enhanced microplane model was combined with Drucker–Prager plasticity in order to show the softening response of concrete [23], while the coupled plastic-damage model was developed in [24] for modelling concrete in the state of unloading and reloading.

Notwithstanding that the established microplane approaches at small strains have been effectively used to describe concrete behaviour, but finite deformations occur in concrete at dynamic cases. In particular, at high confined pressure on tube-squash tests according to experiments conducted in [25], approximately 30–50% of concrete strains were exhibited at deformed shapes with no visible damage or cracks observed. This phenomenon has been supported by numerical simulations using a finite strain microplane damage model in [26], while simulation results obtained by the small strain version showed that the concrete lost almost its entire stiffness. Meanwhile, using softening microplane plasticity at finite strains proposed in [27,28], large plastic deformations were demonstrated for similar cases. Furthermore, strains up to 100% may happen in the state of impact loading due to bored injection piles or anchors, whereas strains achieve approximately 30% while subjected to blast loading or ground shock waves, as mentioned in [25]. Meanwhile, under seismic or earthquake loading, columns of structures were deformed at around 15% of strains until collapse based on [25]. In the case of projectile or missile penetration into a concrete wall, beyond 100% of effective plastic strain occurred [29,30].

Due to all aforementioned circumstances, the small strain microplane model should be extended to the finite strain framework. Hence, the plastic-damage microplane formulation at finite strains regularised by the implicit gradient-enhanced method is proposed within the V-D split. The described model is also equipped with rate dependency to deliver strain rate effects at dynamic cases. Moreover, a coupled contact-adaptive element erosion is implemented herein for the penetration situation. The objective of the present work is to introduce and formulate the rate-dependent plastis-damage microplane model for concrete at finite strains in order to achieve more accurate and reliable responses, particularly at high velocity impact simulations. This work is organised as follows. First, the research motivation is summarised and followed by the proposed constitutive description as well as algorithmic features implemented. Subsequently, numerical examples of impact load cases are simulated and, finally, the results are compared to existing experimental investigations in order to challenge the capability of the newly proposed formulation.

## 2. Material Constitutive Laws at Finite Strains

### 2.1. Microplane Approach with V-D Split

The present work continues to use the V-D split for extending the small strain microplane model to the finite strain regime employing the conjugate strain-stress pair, namely the Green-Lagrange strain tensor and the second Piola-Kirchhoff stress tensor, which is transformed to the Cauchy stress tensor at post-processing [26,28]. Both predecessor models showed that large deformations occur in concrete either at the damaged region or at the plasticity domain. Due to these phenomena, this subsequent work will combine both damage and plasticity within the microplane approach at the finite strain framework using the proposed model in [26,28]. As discussed in [29,31], the Green-Lagrange strain tensor E is chosen to extend the small strain microplane description for concrete to the finite strain formulation computed as

(1) $$E=\frac{1}{2}\left(C-I\right),$$

with the right Cauchy-Green tensor C obtained by the deformation gradient F as follows

(2) $$C=F^{T}F,$$

(3) $$F=\frac{\partial x}{\partial X},$$

where X and x are the initial and spatial Cartesian coordinates, respectively.

Rather than the Biot or Hencky strain tensor, the Green-Lagrange strain tensor is selected since it is the only admissible strain tensor for extending the small strain microplane model to the finite strain formulation due to several reasons. A direct physical interpretation, such as the V-D or N-T decompositions, is clearly explained by the Green-Lagrange strain tensor. Stretch characterisations in the normal strain component should be independent of the stretch in other directions. Similarly, the representation of shear angles in the shear strain component should also be independent of the shear angle in other microplanes as well as the stretch in other directions. Solely the Green-Lagrange strain tensor satisfies these circumstances and, hence, it is chosen to be the strain measure in this extended model.

In general, the V-D decomposition at finite strain theories is multiplicative. Nevertheless, by the fact that the volumetric strain of concrete is always small at all conditions, the multiplicative formulation could be simplified additively for the extension using the Green-Lagrange strain tensor [29,31]. Please note that for other materials with large volume changes, the multiplicative decomposition should be employed instead of the additive split. According to the V-D-T split, the microplane normal strain $E_{N}$ is related to its volumetric and deviatoric parts, $E_{V}$ and $E_{ND}$, as follows

(4) $$E_{N}=E_{V}+E_{ND}\;\mathrm{or}\;E_{ND}=E_{N}-E_{V},$$

where $E_{N}$ and $E_{V}$ are computed as

(5) $$E_{N}=n⊗n:E,$$

(6) $$E_{V}=E_{0}+\frac{1}{2}E_{0}^{2}.$$

While E is obtained from Equation (1) and n denotes the normal vector, $E_{0}$ is described by the Biot strain as $E_{0}=J^{\frac{1}{3}}-1$ with J=detF>0. As the kinematic constraint of the V-D split is used here, the deviatoric component $E_{ND}$ is modified into vector form of the deviatoric strain $E_{D}$

(7) $$E_{D}=E_{ND}n+E_{T},$$

where the tangential strain vector $E_{T}$ is calculated from its third order projection tensor T as

(8) $$E_{T}=T:E,$$

(9) $$T=n\cdot I^{sym}-n⊗n⊗n,$$

with the symmetrical fourth order identity tensor defined as $I^{sym}=\frac{1}{2}\left[I+I^{T}\right]$.

In terms of elastoplasticity, the multiplicative decomposition into elastic and plastic parts is performed using the deformation gradient F [32]. Since concrete undergoes very small elastic strains, the elastic part of the finite strain tensor could be replaced by small strains and it leads non-negative dissipation by plastic strains [29] and, thus, the additive elastic-plastic decomposition is admissible. This approach has been introduced in [28], and it yields accurate plastic behaviour of concrete at finite strains. For hyperelastic materials such as rubbers, the multiplicative formulation should be implemented instead of using the proposed additive approach.

The formulation of microplane models, in general, starts with the macroscopic free-energy function as the integration of its microplane quantities

(10) $$\Psi^{mac}=\frac{3}{4\pi }\int_{\Omega }\Psi^{mic}\left(E\right)\;d\Omega .$$

As seen in Equation (10), the microplane free-energy is the function of the Green-Lagrange strain tensor, its conjugate should be the second Piola-Kirchhoff S stress tensor. Due to material orientations, the stress quantity obtained by applying material laws to the Green-Lagrange strain tensor is the co-rotated Cauchy stress tensor as suggested in [29]. However, the second law of thermodynamics is not satisfied by the non-conjugate pair of the Green-Lagrange strain tensor and the co-rotated Cauchy stress tensor. Besides thermodynamical restrictions, the use of the second Piola-Kirchhoff stress tensor as the stress measure is appropriate for concrete, since this tensor refers to the initial configuration. Concerning the physical meaning of strength and frictional limit within the normal and shear components, the second Piola-Kirchhoff stress tensor is allowed to be the stress measure since no large elastic strain is observed in concrete, yet the finite strain may occur at the inelastic regime. For those reasons, as discussed in [26,28], the approximation of the second Piola-Kirchhoff stress tensor to be equal to the co-rotated Cauchy stress tensor is considered.

By coupling damage and plasticity, the second Piola-Kirchhoff stress tensor S is then computed as

(11) $$S=\frac{3}{4\pi }\int_{\Omega }\left(1-d^{mic}\right)\left[K^{mic}V\left(E_{V}-E_{V}^{p}\right)+2G^{mic}{\mathrm{Dev}}^{T}\cdot \left(E_{D}-E_{D}^{p}\right)\right]\;d\Omega ,$$

where $d^{mic}$ denotes the damage variable separated into compression and tension, while the superscript *p* indicates the plastic quantities. The constants $K^{mic}$ and $G^{mic}$ define the elastic microplane material parameters related to the bulk and shear moduli as $K^{mic}=3K$ and $G^{mic}=G$, respectively. Moreover, the volumetric and deviatoric projection tensors, V and Dev, are obtained as

(12) $$V=\frac{1}{3}J^{\frac{2}{3}}C^{-1},$$

(13) $$\mathrm{Dev}=n\cdot I^{dev}=n\cdot I^{sym}-\frac{1}{3}n\cdot C^{-1}⊗C.$$

Next, the plastic strains evolve following the elastoplastic flow rules

(14) $${\dot{E}}_{V}^{p}=\dot{\lambda }m_{V},\;{\dot{E}}_{D}^{p}=\dot{\lambda }m_{D},$$

where $\dot{\lambda }$ denotes the plastic multiplier, while $m_{V}$ and $m_{D}$ define the flow directions expressed as

(15) $$m_{V}=\frac{\partial F^{mic}}{\partial S_{V}^{e}},\;m_{D}=\frac{\partial F^{mic}}{\partial S_{D}^{e}},$$

where the yield function $F^{mic}$ originates from the Drucker–Prager yield criterion as used in [28], which is now equipped with compression and tension caps. Meanwhile, the superscript *e* indicates the effective stress measure in which $S_{V}^{e}$ and $S_{D}^{e}$ are

(16) $$S_{V}^{e}=K^{mic}\left(E_{V}-E_{V}^{p}\right),\;S_{D}^{e}=2G^{mic}\left(E_{D}-E_{D}^{p}\right).$$

Furthermore, in order to visualise damage and plastic contributions, the scalar values corresponding to the damage variable $d^{mic}$ and the hardening variable $\kappa^{mic}$ are calculated

(17) $$d^{hom}=\frac{\frac{3}{4\pi }\int_{\Omega }d^{mic}d\Omega }{\frac{3}{4\pi }\int_{\Omega }d\Omega },\;\kappa^{hom}=\frac{\frac{3}{4\pi }\int_{\Omega }\kappa^{mic}d\Omega }{\frac{3}{4\pi }\int_{\Omega }d\Omega }.$$

Numerical integrations over the surface of the sphere as found in Equations (10), (11), and (17) are performed using only 21 microplanes reduced from 42 microplanes due to symmetry.

### 2.2. Modified Smooth Three Surface Drucker–Prager Yield Criterion

**Figure 1:**
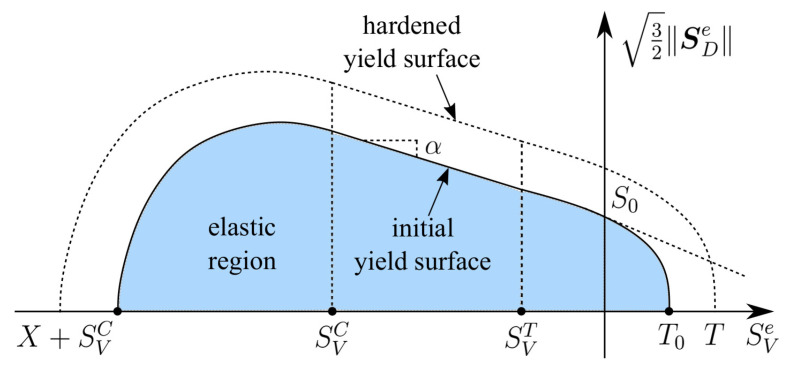
Smooth three-surface yield function as in [24] extended to finite strains.

The smooth cap model within the Drucker–Prager yield criterion is implemented for capturing complex triaxial behaviour of concrete. As discussed in [33], a non-smooth yield surface leads to the singularity in the tangent terms and causes numerical instabilities with slow convergence rates. Accordingly, the smooth yield surface function depicted in Figure 1, based on the study in [34] then implemented in [24], is now extended to the finite strain framework and written in terms of the reference configuration as

(18) $$F^{mic}\left(S_{D}^{e},S_{V}^{e},\kappa \right)=\frac{3}{2}S_{D}^{e}\cdot S_{D}^{e}-F_{1}^{2}\left(S_{V}^{e},\kappa \right)F_{c}\left(S_{V}^{e},\kappa \right)F_{t}\left(S_{V}^{e},\kappa \right).$$

Here, the function $F_{1}$ contains the Drucker–Prager yield criterion with hardening

(19) $$F_{1}=S_{0}-\alpha S_{V}^{e}+F_{h}\left(\kappa \right),$$

with the linear hardening function defined as

(20) $$F_{h}\left(\kappa \right)=D\kappa ,$$

where *D* is the material constant and the hardening variable κ evolves following the flow rule as

(21) $$\dot{\kappa }=\dot{\lambda }.$$

Moreover, the functions $F_{c}$ and $F_{t}$ describe the compression and tension caps, respectively, which are computed as

(22) $$F_{c}=1-H_{c}\left(S_{V}^{C}-S_{V}^{e}\right)\frac{{\left(S_{V}^{e}-S_{V}^{C}\right)}^{2}}{X^{2}},$$

(23) $$F_{t}=1-H_{t}\left(S_{V}^{e}-S_{V}^{T}\right)\frac{{\left(S_{V}^{e}-S_{V}^{T}\right)}^{2}}{{\left(T-S_{V}^{T}\right)}^{2}},$$

where the intersection points of corresponding caps are

(24) $$X=RF_{1}\left(S_{V}^{C}\right),$$

(25) $$T=T_{0}+R_{t}F_{h}\left(\kappa \right).$$

The function $H_{c}$ and $H_{t}$ are defined by the Heaviside function for activating the respective caps as the stress state is within their domains

(26) $$H\left(x\right)=\frac{1}{2}\left(1+\mathrm{sign}(x)\right).$$

Furthermore, in accordance with [35], the calibration of several parameters in the smooth three-surface Drucker–Prager yield function is required in order to simplify the numerical implementation. By knowing concrete data in terms of uniaxial compressive strength $f_{uc}$ in MPa, other empirical formulas can be obtained such as the uniaxial tensile strength $f_{ut}$ and the biaxial compressive strength $f_{bc}$

(27) $$f_{ut}=1.4{\left(f_{uc}/10\right)}^{2/3},\;f_{bc}=1.15f_{uc}.$$

Now, the parameters $S_{0}$ and α in Equation (19) can be determined as

(28) $$\alpha =\frac{\sqrt{3}\left(f_{bc}-f_{uc}\right)}{2f_{bc}-f_{uc}},$$

(29) $$S_{0}=\left(1/\sqrt{3}-\alpha /3\right)f_{uc}.$$

Substituting Equation (27) into Equation (28) yields α=0.2. Moreover, $f_{bc}$ can be related to the yield function as $\sqrt{3/2}∥S_{D}^{e}∥=f_{bc}/\sqrt{3}$ and $S_{V}=-\frac{2}{3}f_{bc}$.

For compression caps as in Equations (22) and (24), the material constant $S_{V}^{C}$ defines the abscissa of the intersection point between the compression cap and the Drucker–Prager yield function, whereas *R* denotes the ratio between the major to the minor axes of the compression cap. The constant $S_{V}^{C}$ is difficult to be determined. However, according to [24], $S_{V}^{C}=-\frac{2}{3}f_{bc}$ can be set as a minimum value mentioned before if no triaxial test data are provided. Meanwhile, for tension caps as in Equations (23) and (25), the material constant $S_{V}^{T}$ defines the abscissa of the intersection point between the tension cap and the Drucker–Prager yield function, whereas $T_{0}$ is the initial intersection point of the tension cap with the volumetric axis. These two parameters are easier to find, and they can also be related to the empirical formula as

(30) $$S_{V}^{T}=-f_{uc}/3,\;T_{0}=f_{ut}/3,$$

while parameter $R_{t}$ controls the increase of the current intersection point *T*. As mentioned in [24], parameter $R_{t}$ can be taken as $R_{t}=1$ if no data for uniaxial cyclic tensile tests are available.

### 2.3. Damage Evolution Law

As mentioned earlier, the damage evolution law in this work is decomposed into compression and tension parts, $d_{c}^{mic}$ and $d_{t}^{mic}$, as proposed in [5] and implemented in [24]

(31) $$1-d^{mic}=\left(1-d_{c}^{mic}\right)\left(1-r_{w}d_{t}^{mic}\right),$$

with the splits

(32) $$d_{c}^{mic}=1-\exp\left(-\beta_{c}\gamma_{c}^{mic}\right),$$

(33) $$d_{t}^{mic}=1-\exp\left(-\beta_{t}\gamma_{t}^{mic}\right),$$

where $\beta_{c}$ and $\beta_{t}$ are the compression and tension damage parameters, respectively, while $r_{w}$ denotes the split weight factor computed as

(34) $$r_{w}=\frac{\sum_{I=1}^{3}\langle E^{I}\rangle }{\sum_{I=1}^{3}\left|E^{I}\right|},$$

where $E^{I}$ is the positive principal value of the Green-Lagrange strain tensor. Moreover, the variables $\gamma_{c}^{mic}$ and $\gamma_{t}^{mic}$ are determined as follows

(35) $$\gamma_{c}^{mic}=\left\{\begin{array}{cc} \eta_{c}^{mic}-\gamma_{c0} & \;\mathrm{for}\;\eta_{c}^{mic}>\gamma_{c0}\\ 0 & \;\mathrm{for}\;\eta_{c}^{mic}\leq \gamma_{c0} \end{array}\right.,$$

(36) $$\gamma_{t}^{mic}=\left\{\begin{array}{cc} \eta_{t}^{mic}-\gamma_{t0} & \;\mathrm{for}\;\eta_{t}^{mic}>\gamma_{t0}\\ 0 & \;\mathrm{for}\;\eta_{t}^{mic}\leq \gamma_{t0} \end{array}\right.,$$

where $\gamma_{c0}$ and $\gamma_{t0}$ denote the damage thresholds for the state of compression and tension, respectively. Meanwhile, the equivalent finite strains $\eta^{mic}$ are obtained in terms of rates as

(37) $${\dot{\eta }}_{c}^{mic}=\left\{\begin{array}{cc} \left(1-r_{w}\right){\dot{E}}_{V}^{p}\; & \mathrm{for}\;{\dot{E}}_{V}^{p}>0\\ 0\; & \mathrm{for}\;{\dot{E}}_{V}^{p}\leq 0 \end{array}\right.,$$

(38) $${\dot{\eta }}_{t}^{mic}=\left\{\begin{array}{cc} r_{w}{\dot{E}}_{V}^{p}\;\;\;\;\; & \mathrm{for}\;{\dot{E}}_{V}^{p}>0\\ 0\;\;\;\;\; & \mathrm{for}\;{\dot{E}}_{V}^{p}\leq 0 \end{array}\right..$$

By activating constraints in Equations (37) and (38), this proposed model covers two conditions such as damage prevention of concrete at high confined pressure loading if ${\dot{E}}_{V}^{p}>0$, and plastic volumetric compaction of concrete in the compression cap if ${\dot{E}}_{V}^{p}\leq 0$.

### 2.4. Rate Dependency

To deliver rate dependency of concrete at dynamic cases, the proposed model is also supplemented by a viscoplastic function, a so-called consistency type formulation, adopted from [36] and applied in [37,38], which is expressed as

(39) $$F_{vp}=\eta_{vp}S_{0}^{2}\dot{\lambda },$$

where $\eta_{vp}$ denotes the viscosity parameter with an arbitrary value. As a consequence, considering rate dependency, Equation (18) yields

(40) $$F^{mic}\left(S_{D}^{e},S_{V}^{e},\kappa ,\dot{\kappa }\right)=\frac{3}{2}S_{D}^{e}\cdot S_{D}^{e}-F_{1}^{2}\left(S_{V}^{e},\kappa ,\dot{\kappa }\right)F_{c}\left(S_{V}^{e},\kappa ,\dot{\kappa }\right)F_{t}\left(S_{V}^{e},\kappa ,\dot{\kappa }\right),$$

where the function $F_{1}$ is modified as

(41) $$F_{1}=S_{0}-\alpha S_{V}^{e}+F_{h}\left(\kappa \right)+F_{vp}\left(\dot{\kappa }\right),$$

and, now, the intersection point *T* increases controlled not only by the parameter $R_{t}$ as in Equation (25) but also by the viscoplastic function $F_{vp}$ as

(42) $$T=T_{0}+R_{t}\left(F_{h}\left(\kappa \right)+F_{vp}\left(\dot{\kappa }\right)\right).$$

**Figure 2:**
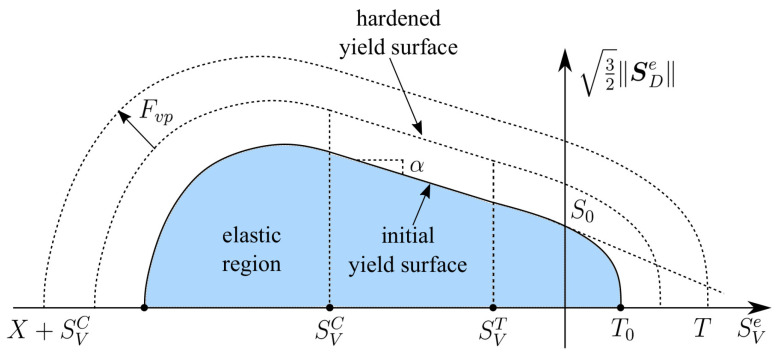
Effect of rate dependency to the yield function.

All functions $F_{1}$, $F_{c}$, and $F_{t}$ as seen in Equation (40) are now a function of $\dot{\kappa }$, which are rate-dependent functions. Furthermore, the effect of rate dependency on the yield function is shown in Figure 2.

### 2.5. Implicit Gradient Enhancement

The microplane approach, which incorporates nonlocal damage or plasticity, or the combination of both, requires a regularisation method to eliminate pathological mesh dependencies and to yield a more stable finite element solution. As implemented successfully in previous models, the present work also employs the so-called implicit gradient-enhanced formulation for regularisation. Two governing equations are then used, such as the balance of linear momentum for the dynamic case

(43) $$\nabla \cdot \sigma +f=\rho \ddot{u},$$

and the modified Helmholtz equation introducing a nonlocal field

(44) $${\bar{\eta }}_{m}-c\nabla^{2}{\bar{\eta }}_{m}=\eta_{m},$$

with its boundary condition

(45) $$\nabla {\bar{\eta }}_{m}\cdot n_{b}=0.$$

In Equation (43), ∇· is the divergence, while σ and f denote the Cauchy stress tensor and the body force vector, respectively. Mass density is defined by ρ, whereas $\ddot{u}$ is the acceleration vector. Moreover, $\nabla^{2}$ and ∇ are the Laplace operator and the gradient, respectively. While the parameter *c* governs the nonlocal interaction range, $n_{b}$ describes the unit normal of outside boundaries. Furthermore, $\eta_{m}$ defines the local variable containing the equivalent strains $\eta_{c}^{mic}$ and $\eta_{t}^{mic}$ for compression and tension, whereas ${\bar{\eta }}_{m}$ is the nonlocal counterpart. For adding only two extra degrees of freedom, according to [24], the variable $\eta_{m}$ is taken into consideration as

(46) $$\eta_{m}=\left[\begin{array}{c} \eta_{mc}\\ \eta_{mt} \end{array}\right]=\left[\begin{array}{c} \frac{1}{4\pi }\int_{\Omega }\eta_{c}^{mic}d\Omega \\ \frac{1}{4\pi }\int_{\Omega }\eta_{t}^{mic}d\Omega \end{array}\right].$$

Nonetheless, as found in [39,40], the over-nonlocal method can achieve a full regularisation in plastic-damage descriptions. Therefore, the equivalent strains $\eta_{c}^{mic}$ and $\eta_{t}^{mic}$ are enhanced as

(47) $${\hat{\eta }}_{c}^{mic}=m{\bar{\eta }}_{mc}+\left(1-m\right)\eta_{c}^{mic},$$

(48) $${\hat{\eta }}_{t}^{mic}=m{\bar{\eta }}_{mt}+\left(1-m\right)\eta_{t}^{mic}.$$

It is stated that localisation still occurs at the strain softening regime if the value m=1 is applied, which means the standard regularisation is implemented. Hence, the value of *m* should be taken as larger than 1 to eliminate localisation and achieve regularisation. Accordingly, $\eta_{c}^{mic}$ and $\eta_{t}^{mic}$ in Equations (35) and (36) are now replaced by ${\hat{\eta }}_{c}^{mic}$ and ${\hat{\eta }}_{t}^{mic}$.

## 3. Algorithmic Aspects

### 3.1. Finite Element Formulation

As standard procedure, the weight functions δu and $\delta {\bar{\eta }}_{m}$ are used for obtaining the weak forms of Equations (43) and (44)

(49) $$\int_{B_{t}}\delta u\cdot \nabla \cdot \sigma \;dv+\int_{B_{t}}\delta u\cdot f\;dv=\int_{B_{t}}\delta u\cdot \rho \ddot{u}\;dv,$$

(50) $$\int_{B_{t}}\delta {\bar{\eta }}_{m}{\bar{\eta }}_{m}\;dv-\int_{B_{t}}\delta {\bar{\eta }}_{m}c\nabla^{2}{\bar{\eta }}_{m}\;dv=\int_{B_{t}}\delta {\bar{\eta }}_{m}\eta_{m}\;dv.$$

With the help of the Gauss theorem and the Cauchy theorem as well as the boundary condition in Equation (45), Equations (49) and (50) become

(51) $$\int_{\partial B_{t}}t\cdot \delta u\;da+\int_{B_{t}}\delta u\cdot f\;dv=\int_{B_{t}}\rho \delta u\cdot \ddot{u}\;dv+\int_{B_{t}}\sigma :\nabla \delta u\;dv,$$

(52) $$\int_{B_{t}}\delta {\bar{\eta }}_{m}{\bar{\eta }}_{m}\;dv+\int_{B_{t}}\nabla \delta {\bar{\eta }}_{m}c\nabla {\bar{\eta }}_{m}=\int_{B_{t}}\delta {\bar{\eta }}_{m}\eta_{m}\;dv.$$

For carrying out the spatial discretisation using shape functions N, the displacement and the variational field as well as their gradients are employed

(53) u=Nd,δu=Nδd,

(54) $$\nabla u=\frac{\partial N}{\partial x}d,\;\;\nabla \delta u=\frac{\partial N}{\partial x}\delta d.$$

Analogously, the discretisation for the nonlocal field using the nonlocal shape function $\bar{N}$ is considered as

(55) $${\bar{\eta }}_{m}=\bar{N}\;\bar{E},\;\;\;\delta {\bar{\eta }}_{m}=\bar{N}\delta \bar{E},$$

(56) $$\nabla {\bar{\eta }}_{m}=\frac{\partial \bar{N}}{\partial x}\bar{E},\;\nabla \delta {\bar{\eta }}_{m}=\frac{\partial \bar{N}}{\partial x}\delta \bar{E},$$

where d and $\bar{E}$ denote nodal displacements and nodal nonlocal equivalent finite strains, respectively.

The discretisation using the above equations and the linearisation for Equations (51) and (52) should be used in order to be solved using an iterative Newton-Raphson method. Now, the coupled linearised system is obtained as

(57) $$R=\left[\begin{array}{c} R_{u}\\ R_{\bar{\eta }} \end{array}\right]=\left[\begin{array}{c} f_{u}^{ext}\\ f_{\bar{\eta }}^{ext} \end{array}\right]-\left[\begin{array}{c} f_{u}^{int}\\ f_{\bar{\eta }}^{int} \end{array}\right],$$

(58) $$\left[\begin{array}{cc} S_{uu,i} & K_{u\bar{\eta },i}\\ K_{\bar{\eta }u,i} & K_{\bar{\eta }\bar{\eta },i} \end{array}\right]\left[\begin{array}{c} \Delta d_{,i+1}\\ \Delta {\bar{E}}_{,i+1} \end{array}\right]=-\left[\begin{array}{c} R_{u,i}\\ R_{\bar{\eta },i} \end{array}\right],$$

where the residual vectors for the mechanical and the nonlocal parts are

(59) $$R_{u,i}=\int_{B_{t}}N^{T}\rho N\;dv\cdot \ddot{d}+\int_{B_{t}}\frac{\partial N}{\partial x}\sigma \;dv-\int_{\partial B_{t}}N^{T}t\;da-\int_{B_{t}}N^{T}f\;dv,$$

(60) $$R_{\bar{\eta },i}=\int_{B_{t}}\frac{\partial {\bar{N}}^{T}}{\partial x}c\frac{\partial \bar{N}}{\partial x}\bar{E}\;dv+\int_{B_{t}}{\bar{N}}^{T}\left[\bar{N}\;\bar{E}-\eta_{m}\right]\;dv.$$

The submatrix $S_{uu,i}$ in Equation (58) is evaluated by the Newmark transient solver to analyse dynamic cases as

(61) $$S_{uu,i}=M\frac{1}{\beta_{n}\Delta t^{2}}+K_{uu,i},$$

where $\beta_{n}$ and Δt denote the Newmark parameter and the incremental time step, respectively. Moreover, the mass matrix M and the submatrix $K_{uu,i}$ are

(62) $$M=\int_{B_{t}}N^{T}\rho N\;dv,$$

(63) $$K_{uu}=\int_{B_{t}}\frac{\partial N^{T}}{\partial x}c\frac{\partial N}{\partial x}\;dv+\int_{B_{t}}\frac{\partial N}{\partial x}\sigma \frac{\partial N^{T}}{\partial x}I\;dv.$$

The other submatrices in Equation (58) are then

(64) $$K_{u\bar{\eta },i}=\int_{B_{t}}\frac{\partial N^{T}}{\partial x}\frac{\partial \sigma }{\partial {\bar{\eta }}_{m}}\bar{N}\;dv,$$

(65) $$\begin{array}{cc} K_{\bar{\eta }u,i} & =\int_{B_{t}}{\bar{N}}^{T}\left({\bar{\eta }}_{m}-\eta_{m}\right)\frac{\partial N}{\partial x}\;dv-\int_{B_{t}}{\bar{N}}^{T}\frac{\partial \eta_{m}}{\partial E}I^{sym}\frac{\partial N}{\partial x}\;dv-\int_{B_{t}}c\left(\frac{\partial N^{T}}{\partial x}\frac{\partial N}{\partial x}\right)\frac{\partial {\bar{\eta }}_{m}}{\partial x}\;dv\\ \; & +\int_{B_{t}}c\left(\frac{\partial N^{T}}{\partial x}\frac{\partial {\bar{\eta }}_{m}}{\partial x}\right)\frac{\partial N}{\partial x}\;dv+\int_{B_{t}}c\frac{\partial N^{T}}{\partial x}\left(\frac{\partial {\bar{\eta }}_{m}}{\partial x}\frac{\partial N}{\partial x}\right)\;dv, \end{array}$$

(66) $$K_{\bar{\eta }\bar{\eta },i}=\int_{B_{t}}{\bar{N}}^{T}\bar{N}\;dv+\int_{B_{t}}\frac{\partial {\bar{N}}^{T}}{\partial x}c\frac{\partial \bar{N}}{\partial x}\;dv.$$

All required derivations for the tangent terms can be found in Appendix A.

### 3.2. Stress Return Algorithm

Two conditions may occur, either the stress state is still in the elastic region, which lies inside the yield surface, or the stress state enters the plastic regime, which is outside the yield surface. If the stress fulfils the yield criterion $F^{mic}\leq 0$, the condition is elastic. Therefore, all plastic strains at the current time step are equal to the condition at the previous time step as

(67) $$E_{V,n+1}^{p}=E_{V,n}^{p},\;E_{D,n+1}^{p}=E_{D,n}^{p},\;\Delta \lambda_{n+1}=0,$$

where the subscript n+1 indicates the current time step, while the subscript *n* denotes the previous one. Then, the evaluation of the trial stresses and the yield function denoted by the subscript tr are

(68) $$S_{V,tr}^{e}=K^{mic}\left(E_{V}-E_{V,n}^{p}\right),$$

(69) $$S_{D,tr}^{e}=2G^{mic}\left(E_{D}-E_{D,n}^{p}\right),$$

(70) $$F_{tr}^{mic}=\frac{3}{2}S_{D,tr}^{e}\cdot S_{D,tr}^{e}+F_{1}^{2}\left(S_{V,tr}^{e},\kappa_{n},{\dot{\kappa }}_{n}\right)F_{c}\left(S_{V,tr}^{e},\kappa_{n},{\dot{\kappa }}_{n}\right)F_{t}\left(S_{V,tr}^{e},\kappa_{n},{\dot{\kappa }}_{n}\right).$$

The return algorithm needs to be enforced as $F^{mic}=0$ if the yield function is not fulfilled, which means that the step enters the plastic condition

(71) $$F^{mic}=\frac{3}{2}S_{D,n+1}^{e}\cdot S_{D,n+1}^{e}-F_{1}^{2}F_{c}F_{t}=0,$$

(72) $$S_{V,n+1}^{e}=S_{V,tr}^{e}-\Delta \lambda_{n+1}K^{mic}m_{V},$$

(73) $$S_{D,n+1}^{e}=S_{D,tr}^{e}-\Delta \lambda_{n+1}2G^{mic}m_{D},$$

(74) $$\kappa_{n+1}=\kappa_{tr}+\Delta \lambda_{n+1}.$$

For rate dependency, the evolution of $\dot{\kappa }$ is simply as

(75) $${\dot{\kappa }}_{n+1}=\frac{\Delta \lambda_{n+1}}{\Delta t}.$$

Using Equation (15), the flow directions $m_{V}$ and $m_{D}$ are obtained as follows

(76) $$m_{V}=-2F_{1}\frac{\partial F_{1}}{\partial S_{V}^{e}}F_{c}F_{t}-F_{1}^{2}\frac{\partial F_{c}}{\partial S_{V}^{e}}F_{t}-F_{1}^{2}F_{c}\frac{\partial F_{t}}{\partial S_{V}^{e}},$$

(77) $$m_{D}=3S_{D}^{e}.$$

Substituting Equation (77) into Equation (73) yields

(78) $$S_{D,n+1}^{e}=S_{D,tr}^{e}-6\Delta \lambda_{n+1}G^{mic}S_{D,n+1}^{e}.$$

As can be seen from Equation (78), $S_{D,n+1}^{e}$ and $S_{D,tr}^{e}$ are collinear and one can rewrite Equation (78) as

(79) $$∥S_{D,n+1}^{e}∥=∥S_{D,tr}^{e}∥-6\Delta \lambda_{n+1}G^{mic}∥S_{D,n+1}^{e}∥.$$

Then, substituting Equation (79) into Equation (71) results in

(80) $$F^{mic}=\frac{3}{2}\frac{∥S_{D,tr}^{e}{∥}^{2}}{{\left(1+6\Delta \lambda_{n+1}G^{mic}\right)}^{2}}-F_{1}^{2}F_{c}F_{t}.$$

For obtaining the plastic multiplier $\Delta \lambda_{n+1}$ and the volumetric stress $S_{V,n+1}^{e}$, Equation (80) can be solved simultaneously with the help of Equation (72), so that

(81) $$F_{\lambda }=\frac{3}{2}\frac{∥S_{D,tr}^{e}{∥}^{2}}{{\left(1+6\Delta \lambda_{n+1}G^{mic}\right)}^{2}}-F_{1}^{2}F_{c}F_{t},$$

(82) $$F_{V}=S_{V,n+1}^{e}-S_{V,tr}^{e}+\Delta \lambda_{n+1}K^{mic}m_{V}.$$

By deriving both Equations (81) and (82) with respect to $\Delta \lambda_{n+1}$ and $S_{V,n+1}^{e}$, one obtains

(83) $$\frac{\partial F_{\lambda }}{\partial \Delta \lambda_{n+1}}=-18G^{mic}\frac{∥S_{D,tr}^{e}{∥}^{2}}{{\left(1+6\Delta \lambda_{n+1}G^{mic}\right)}^{3}}+\frac{\partial F^{mic}}{\partial \Delta \lambda_{n+1}},$$

(84) $$\frac{\partial F_{\lambda }}{\partial S_{V,n+1}^{e}}=m_{V},$$

(85) $$\frac{\partial F_{V}}{\partial \Delta \lambda_{n+1}}=K^{mic}m_{V}+\Delta \lambda_{n+1}K^{mic}\frac{\partial m_{V}}{\partial \Delta \lambda_{n+1}},$$

(86) $$\frac{\partial F_{V}}{\partial S_{V,n+1}^{e}}=1+\Delta \lambda_{n+1}K^{mic}\frac{\partial m_{V}}{\partial S_{V,n+1}^{e}}.$$

Subsequently, the updated deviatoric stress and plastic strains are

(87) $$S_{D,n+1}^{e}=\frac{S_{D,tr}^{e}}{\left(1+6\Delta \lambda_{n+1}G^{mic}\right)},$$

(88) $$E_{D,n+1}^{p}=E_{D,n+1}-\frac{S_{D,n+1}^{e}}{2G^{mic}},$$

(89) $$E_{V,n+1}^{p}=E_{V,n+1}-\frac{S_{V,n+1}^{e}}{K^{mic}}.$$

## 4. Penetration Model

### 4.1. Contact Mechanism

To limit the discussion here, a general node-to-node contact mechanism is applied and it is briefly explained. This contact model is simply used since forces are computed directly at the nodes, and it provides a straightforward procedure to find the contact nodes. Moreover, a frictionless model is implemented so that only normal components of contact forces are considered, which implies that the tangential components are negligible. Since the contact model is not the main focus in the present work, no special properties are provided so that general codes for contact models can be used herein. Slight modifications are conducted in order to exclude contact nodes in damaged elements when the contact model is coupled to the adaptive element erosion procedure.

### 4.2. Adaptive Element Erosion

**Figure 3:**
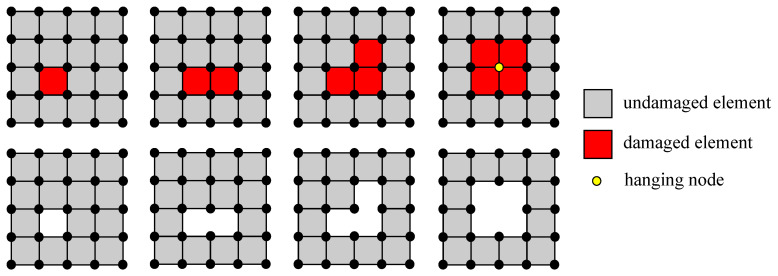
Illustration of adaptive element erosion scheme.

As mentioned earlier, damaged elements should be removed if they are destroyed and the impactor may penetrate into the concrete structure. To accommodate this feature, so-called adaptive element erosion is introduced in the present work supplementing the proposed material models. Here, the elimination method is implemented in a straightforward scheme as shown in Figure 3. A certain damage value as failure threshold is firstly determined prior to start the removal procedure as

(90) $$\left\{\begin{array}{cc} \mathrm{damaged} & \;\mathrm{for}\;d^{hom}\geq d^{val}\\ \mathrm{undamaged} & \;\mathrm{for}\;d^{hom}<d^{val} \end{array}\right.,$$

where $d^{hom}$ is a scalar damage measure which is homogenised from all microplanes as computed using Equation (17), while $d^{val}$ is determined as the critical value.

From the material level, the value of $d^{hom}$ is obtained, then it is looped over Gauss points on the element level. Here, an 8-node brick element in the three-dimensional model is used, so that eight Gauss points are taken into account. If $d^{val}$ is achieved in all eight Gauss points, one element is completely damaged and it can be considered to be a failed element indicated by the red-coloured square in Figure 3. Since the stiffness of the damaged element has been degraded up to almost zero, the element is now inactive, and it has to be excluded from the finite element computation without re-meshing. Hence, only active elements are involved in the computation. Furthermore, a special treatment is performed only if all neighbouring elements of a node are completely damaged. This condition causes the node connecting to its surrounding elements being unsupported and it becomes a hanging node, denoted by the yellow-coloured circle as shown in Figure 3. Now, this node is also skipped and the profile is reconstructed without re-meshing as previously.

**Figure 4:**
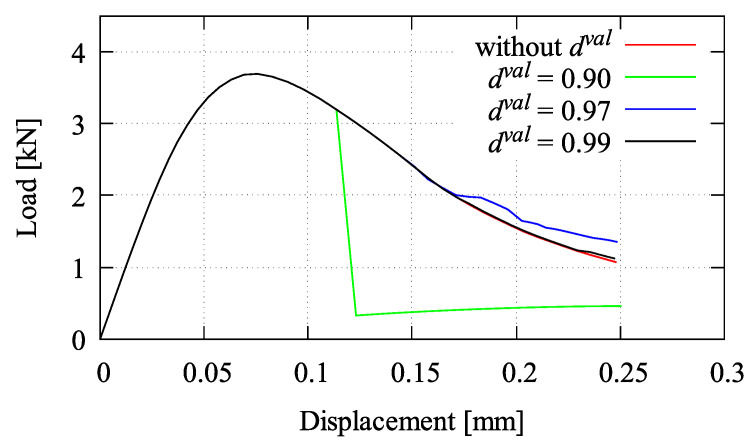
Load-displacement relations for different damage values $d^{val}$ in four-point bending test.

To examine the adaptive element erosion, a four-point bending test at static loading is simulated. The proposed gradient-enhanced plastic-damage microplane model is used for modelling concrete herein. To evaluate and analyse the effect of the damage value tolerance $d^{val}$ to concrete responses, the values of 0.9, 0.97, and 0.99 are chosen. As can be seen in Figure 4, a good approximation is achieved by $d^{val}=0.99$ which coincides to the curve without $d^{val}$, while the simulation with $d^{val}=0.90$ gives a different response among others. Using $d^{val}=0.90$, the stiffness is degraded too early and, thus, it leads to the spurious damage since the undamaged elements may categorise as damaged elements.

**Figure 5:**
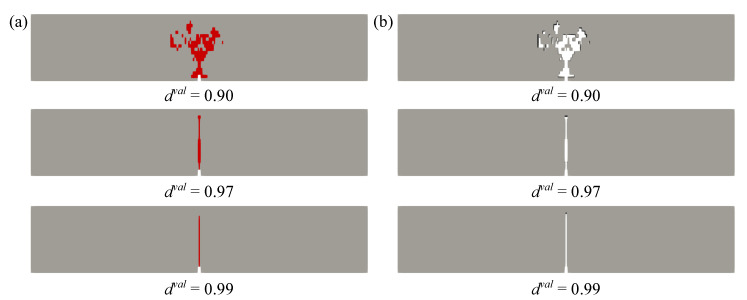
Four-point bending test using three different $d^{val}$ implementing an adaptive element erosion: (**a**) crack paths and (**b**) removed elements.

Moreover, the validation continues to the visualisation of crack paths at final step of the bending test using those three different damage values $d^{val}$ as seen in Figure 5a, whereas Figure 5b visualises the removed elements of the corresponding crack paths. Analogously, using $d^{val}=0.90$, the crack path becomes unrealistic since spurious damage may occur as explained before. Therefore, based on these investigations, $d^{val}=0.99$ is implemented in the present work so that Equation (90) reads

(91) $$\left\{\begin{array}{cc} \mathrm{damaged} & \;\mathrm{for}\;d^{hom}\geq 0.99\\ \mathrm{undamaged} & \;\mathrm{for}\;d^{hom}<0.99 \end{array}\right..$$

**Figure 6:**
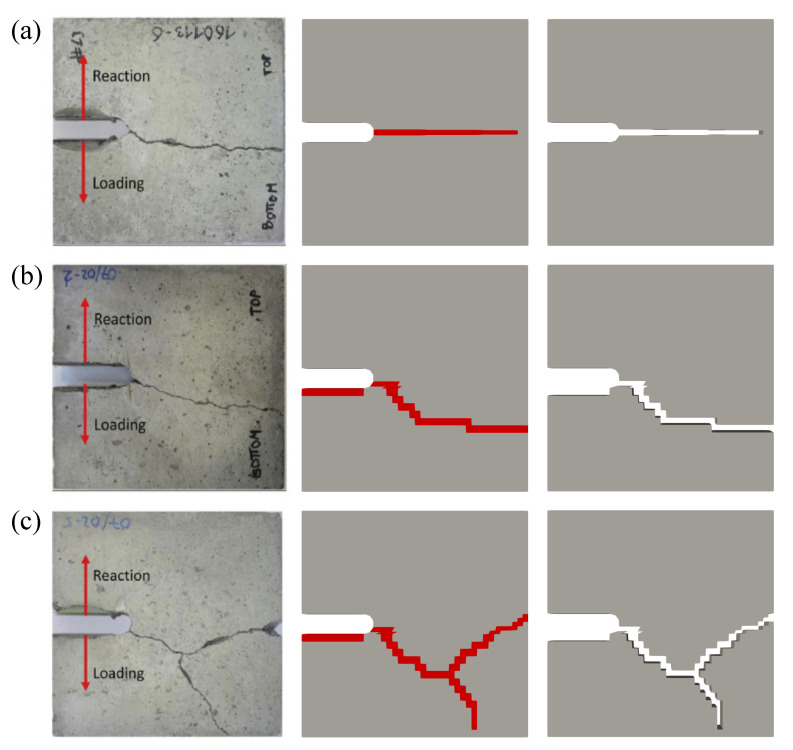
Simulation of compact tension specimens displaying crack paths and removed elements compared to experiments in [41] at three different velocities: (**a**) 0.035 m/s, (**b**) 1.4 m/s, and (**c**) 4.3 m/s.

Another example is a compact tension test in order to observe that the proposed adaptive element erosion also works well for crack branching. The simulations with three different velocities of 0.035 m/s, 1.4 m/s, and 4,3 m/s are performed according to [26] based on experiments in [41] using $d^{val}=0.99$, as obtained before. Figure 6a displays the crack paths of the compact tension specimens, while Figure 6b depicts the removed elements corresponding to its crack paths.

### 4.3. Coupled Contact and Adaptive Element Erosion

Since the final goal of this work is to simulate high velocity impact on concrete plates, the contact model needs to be combined with adaptive element erosion introduced previously. Therefore, the contact mechanism should be applied when the impactor hits the concrete plate surface, whereas the adaptive element erosion is used to eliminate damaged elements as the impactor may penetrate into the structure.

**Figure 7:**
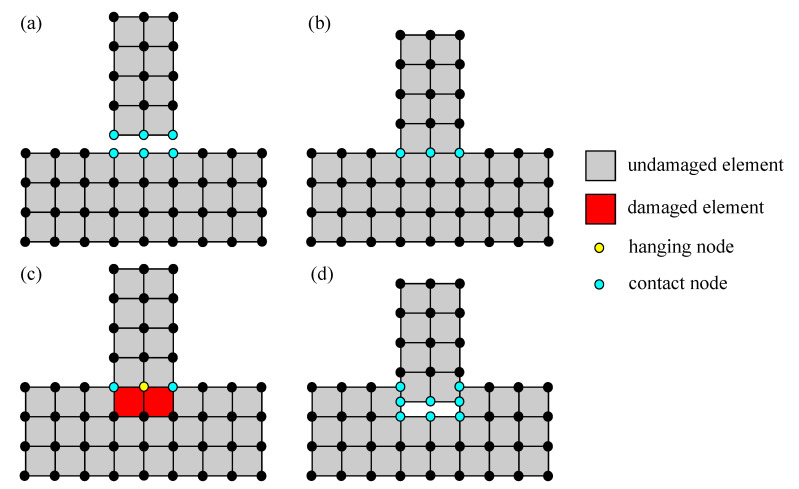
Coupled contact mechanism and adaptive element erosion at impact cases: (**a**) initial condition, (**b**) contact process, (**c**) detection of damaged elements during contact, (**d**) searching of new contact nodes after removing elements.

An initial condition with a gap between an impactor and concrete plate is shown schematically in Figure 7a, while the contact process starts when the impactor hits the plate surface as in Figure 7b and contact nodes are indicated by blue-coloured circles. The regular contact mechanism works during this process. If some elements are damaged as explained before, these elements are then eliminated and a hanging node can probably exist, see Figure 7c. Once the unsupported node is also removed, the impactor continues to move down following the applied velocity and the new contact nodes are found automatically based on the gap tolerance value as in the beginning process, see also Figure 7d. Hence, the penetration process continues and the next step proceeds repeatedly up to failure.

## 5. Model Parameters Adjustment

Several types of model parameter are investigated by numerical examples performed in here, i.e., parameters for elasticity, plasticity, damage, and nonlocal features. Young’s modulus *E* and Poisson’s ratio ν as the elasticity parameters are simply taken from the experimental data. Next, the plasticity parameters in this proposed model consist of $f_{uc}$, $S_{V}^{C}$, *R*, $R_{t}$, and *D*. The uniaxial compressive strength $f_{uc}$ is mostly given in the experimental data, whereas the parameter *R* can be approximated by $R=X_{0}/f_{1}\left(S_{V}^{C}\right)$ as mentioned in [24]. Meanwhile, the hardening *D* is related to the damage parameters, hence, it needs to be identified corresponding to $\beta_{c}$ and $\gamma_{c0}$ by performing a uniaxial cyclic compression test, whereas $R_{t}$ is related to the damage parameter $\beta_{t}$ and identified by carrying out a uniaxial cyclic tension test. As a starting point, $R_{t}=1$ and $\beta_{t}≃1.5\beta_{c}$ can be considered, see [24]. Moreover, due to the fact that softening in the tensile state occurs right away subsequent to the elastic limit, the tension damage threshold is considered to be $\gamma_{t0}=0$ in all simulations.

Moreover, the nonlocal parameter *c* is quite challenging to be identified as $c=l^{2}$. Nevertheless, several attempts were conducted in [42,43,44] to find the parameter *c*. For the parameter *m*, as explained earlier, it should be taken larger than 1 to achieve a full regularisation. According to [45], the value of *m* is considered to be 1<m≤1.1, while [40] used the value slightly beyond 1 as m=1.005. However, m=2.5 is used in all simulations herein. For rate dependency, an arbitrary value larger than zero of the viscosity parameter $\eta_{vp}$ gives a rate effect in the modelling. A benefit of the consistency type formulation in the viscoplastic model as in Equation (39) is to consider the constitutive law with or without strain rate effects in a straightforward manner. To produce a rate-independent model, $\eta_{vp}=0$ can be simply taken, whereas in order to activate the rate-sensitive formulation, $\eta_{vp}=1$ s/MPa is implemented in all subsequent simulations.

### 5.1. Four-Point Bending Test at Cyclic Loading

**Figure 8:**
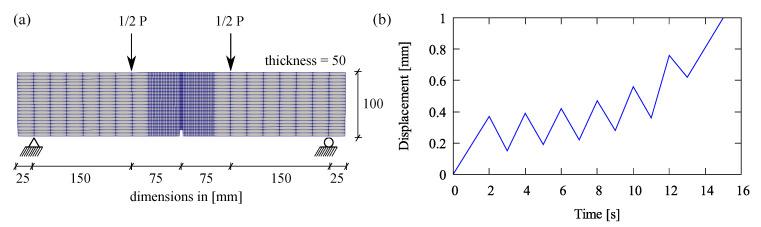
Four-point bending test: (**a**) geometry and (**b**) applied cyclic displacement.

**Figure 9:**
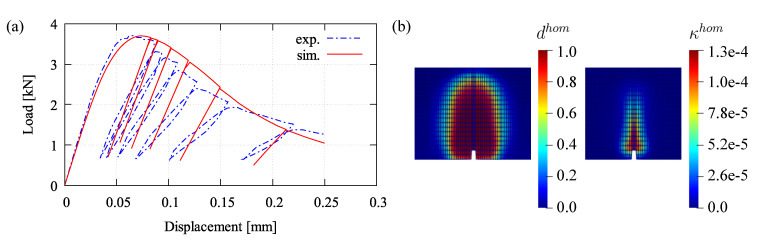
Four-point bending test result: (**a**) load-displacement relation and (**b**) damage and plastic distribution, $d^{hom}$ and $\kappa^{hom}$.

**Figure 10:**
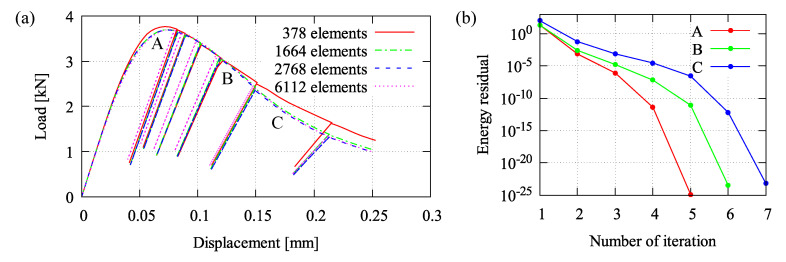
Four-point bending simulation: (**a**) comparison of different mesh densities and (**b**) accompanying convergence rates for mesh with 2768 elements.

A four-point bending test at cyclic loading is simulated. This analysis is performed to identify the material parameters which need to be fitted with respect to experimental data in [46]. The meshed beam with a notch including boundary conditions and applied cyclic loading for the bending simulation is provided in Figure 8a,b, respectively. Table 1 provides all model parameters used in this bending test simulation. A load-displacement relation obtained by the proposed formulation using the fitted parameters is plotted in Figure 9a, while damage and plastic contributions computed by Equation (17) are depicted in Figure 9b. Moreover, mesh insensitivity of the proposed model is achieved as plotted in Figure 10a with accompanying convergence rates for the mesh with 2464 elements at chosen certain time steps shown in Figure 10b.

**Figure 11:**
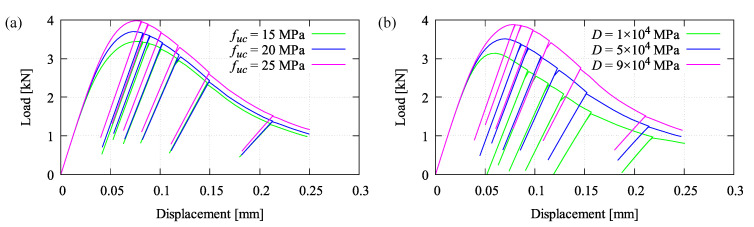
Effect of parameters with respect to the load-displacement relation: (**a**) compressive strength $f_{uc}$ and (**b**) hardening stiffness *D*.

Two model parameters, for instance the uniaxial compressive strength $f_{uc}$ and the hardening stiffness *D*, are observed to figure out their effects with respect to the load-displacement response. Three different values are arbitrarily chosen to perform the simulation. As can be seen in Figure 11a, the obvious influence of $f_{uc}$ is shown that increasing $f_{uc}$ affects the higher peak load. This parameter is simply taken from the experiment or, if no data provided, the value can be empirically determined corresponding to the Young’s modulus *E* as considered in many building codes. Meanwhile, the parameter *D* can be identified relating to other damage parameters, $\beta_{c}$ and $\gamma_{c0}$, as mentioned previously. However, its effect to the hardening-softening branch of the load-displacement curve can be seen in Figure 11b. The curve slope downgrades significantly by using the lower value of *D*, otherwise the higher *D* leads to the higher hardening and the delayed softening so that the curve is going down slowly.

### 5.2. Homogeneous Test

**Figure 12:**
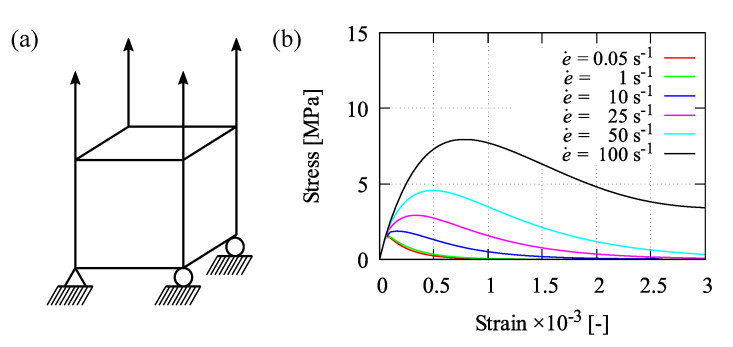
Homogeneous test: (**a**) illustration of uniaxial tension test and (**b**) strain rate effect.

**Figure 13:**
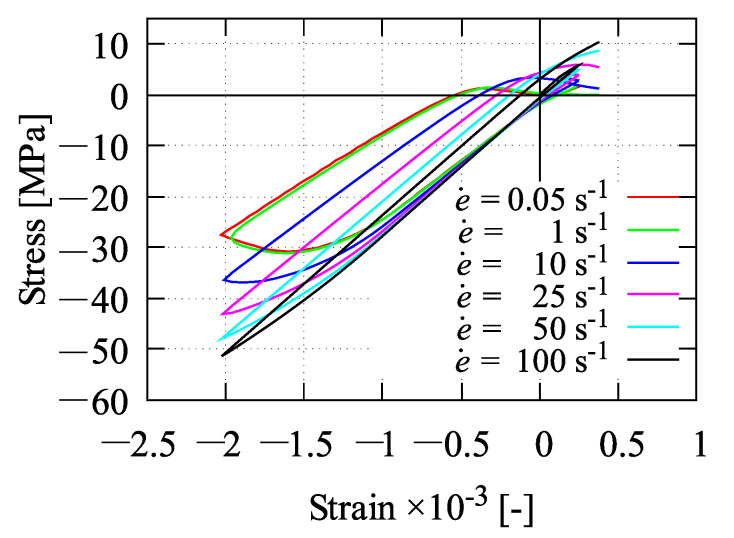
Homogeneous test at cyclic loading with different strain rates.

In addition to identify the damage parameters *D*, $\beta_{c}$, and $\gamma_{c0}$ by simulating the uniaxial compression test as well as the parameters $\beta_{t}$ and $R_{t}$ by performing the uniaxial tension test, a homogeneous test can also be used to find out about the effect of strain rates $\dot{e}$ as shown in Figure 12. Only one element is simulated subjected to uniaxial tension as illustrated in Figure 12a. The activation of strain rate effects is simply performed by taking $\eta_{vp}=1$ s/MPa as mentioned previously. Here, six strain rates of 0.05 s${}^{-1}$, 1 s${}^{-1}$, 10 s${}^{-1}$, 25 s${}^{-1}$, 50 s${}^{-1}$, and 100 s${}^{-1}$ are chosen to demonstrate the influence of the proposed rate-dependent model. Due to the applied strain rates, one can see the growth of peak stresses in terms of stress-strain relations plotted in Figure 12b. In other words, by increasing the strain rate, the stress-strain curve is getting higher accordingly. Furthermore, the effect of different strain rates at cyclic uniaxial tension-compression test with respect to the stress-strain response is depicted in Figure 13.

## 6. Numerical Examples

A couple of concrete plate simulations with different thickness subjected to high velocity impacts are carried out using the newly proposed framework. Comparisons between the small and finite strain model simulated by the same material parameters are provided and compared also to the experimental data.

### 6.1. Thick Plate Simulation

**Figure 14:**
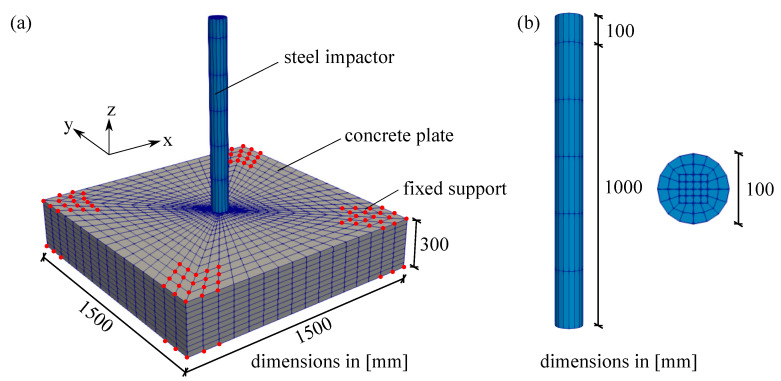
Finite element mesh of thick plate impact test: (**a**) geometry and (**b**) dimension of the steel impactor.

First, an example of a thick plate is simulated to show the penetration of an impactor into the concrete plate. As reported in [47], a machine plate of 540 kg and a steel impactor of 50 kg at a height of 2 m hit a concrete plate with the dimensions of 1500 × 1500 × 300 mm. The impactor velocity is considered to be 25,000 mm/s according to [26]. Furthermore, as boundary conditions, the concrete plate is restrained at all four corners denoted by red dots as can be seen in Figure 14. This impact test is modelled using one-fourth of the whole specimen by 5241 nodes and 3272 elements due to the symmetrical condition. The whole geometry of the thick plate impact test in finite element mesh discretisation is depicted in Figure 14a, while the dimension of its impactor is shown in Figure 14b. All material properties of this impact test are provided in Table 2.

**Figure 15:**
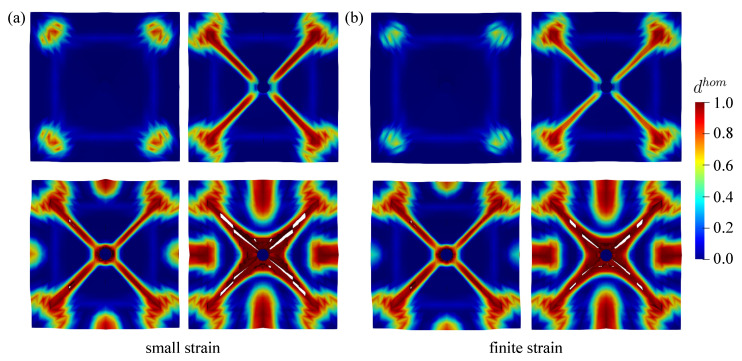
Top view of damage evolution for thick plate impact test: (**a**) small strain and (**b**) finite strain simulation.

**Figure 16:**
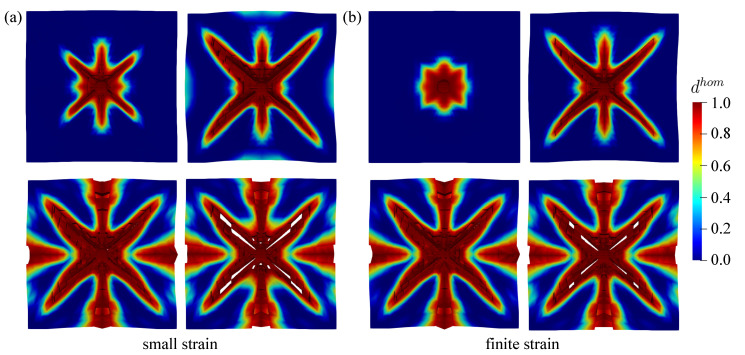
Bottom view of damage evolution for thick plate impact test: (**a**) small strain and (**b**) finite strain simulation.

**Figure 17:**
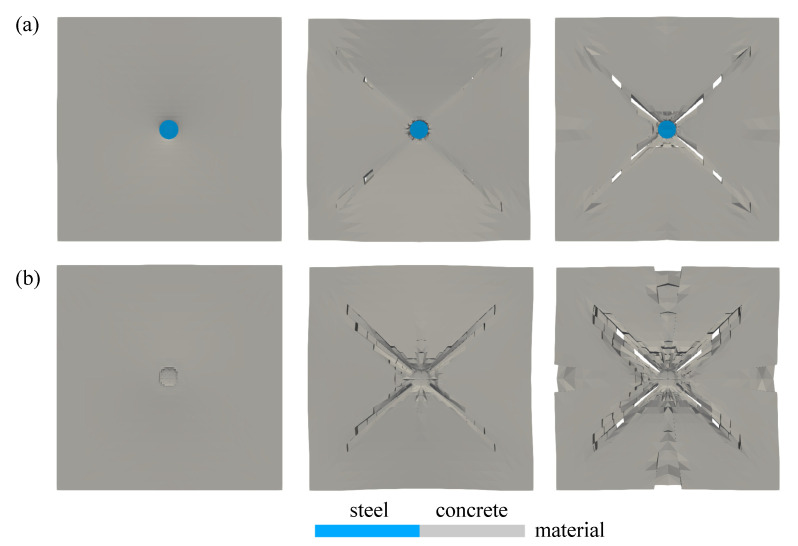
Crack growth visualisation of thick plate impact test: (**a**) top and (**b**) bottom view.

The proposed plastic-damage microplane model at finite strains including the contact mechanism coupled to the adaptive element erosion is able to represent the damage evolution including the eroded elements as shown in Figure 15a,b seen from top view of the plate surface at the chosen time step for small and finite strain simulation, respectively. Meanwhile, the visualisation of damage evaluation from the bottom side for both models is depicted in Figure 16a,b. As explained before, the damage value tolerance $d^{val}$ is considered to be 0.99, which means that the element is removed once the value of $d^{hom}$ in all eight Gauss points of one element is equal or larger than 0.99. From both Figure 15 and Figure 16, one can see the difference between the small and finite formulation that the damage of concrete plate at small strains evolves faster than the damage in finite strain simulations. It means that the faster degradation of material stiffness occurs in numerical simulations using the small strain formulation rather than using the finite strain model. Moreover, in order to display a more realistic crack growth, Figure 17 represents only the material view of steel and concrete, which is simulated by the finite strain model.

As shown in Figure 17, crack initiation starts immediately on the mid-bottom side of the concrete plate once the steel impactor hits the plate surface. Further cracks propagate diagonally towards the four fixed supports implemented in each plate corner. Crack openings get wider following the impactor penetration into the concrete plate. By these results, the use of damage values obtained from the proposed penetration model has an advantage since it is able to be coupled with the element erosion in a straightforward manner. A good visualisation of the crack growth is valuable to supplement the proposed material model, which has indeed yielded meaningful results without coupling the element erosion method altogether.

**Figure 18:**
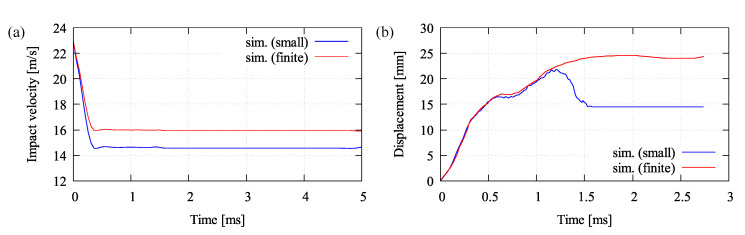
Simulation results comparing small and finite strain model: (**a**) velocity-time and (**b**) displacement-time relation.

Besides the damage evolution, the velocity-time and displacement-time relations are also plotted to show the differences between the small and finite strain microplane model. Analogous to the previous explanation regarding the material stiffness degradation, the velocity and displacement responses differ accordingly. The impact velocity is observed at a node located on the contact surface, while the displacement is investigated on the mid-bottom side of the plate. As one can see in Figure 18a, the impact velocity simulated by the small strain model drops immediately, so that the finite strain result has a higher residual velocity, which differs approximately 2.3 m/s. Likewise, since the material stiffness of the small strain model degrades faster as explained earlier, its displacement also deviates accordingly as shown in Figure 18b. Here, more or less 10 mm difference of the displacement is discovered between the small and finite strain simulation result.

**Figure 19:**
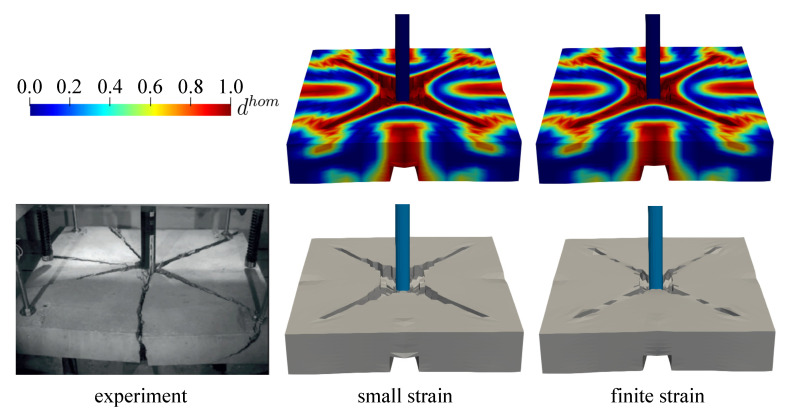
Comparison of damaged plate reported in [47] to both small and finite strain models.

**Figure 20:**
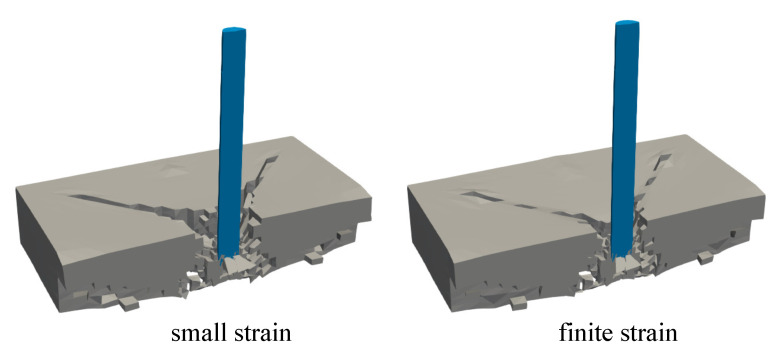
Cross-section view of damaged plate for small and finite strain models.

In this simulation, only a picture of damaged plate is compared to the simulation results since no other experimental data are reported. The comparison for both small and finite strain models to the experimental investigation is depicted in Figure 19. Some deviations of the crack propagation direction obtained by the experiment may occur due to the heterogeneity of material constituents in concrete, for instance diverse aggregate sizes as well as porosity between aggregates and cement paste. However, the numerical result is able to yield quite similar crack patterns compared to the experimental investigation. From Figure 20, one can see that more damage occurs surrounding the impactor in the small strain simulation, whereas the finite strain model gives a closer result to the experimental data. This observed inaccuracy may indicate that a finite strain case happens in the present impact test, so that the small strain formulation is not sufficient for modelling this phenomenon.

### 6.2. Small Thin Plate Simulation

**Figure 21:**
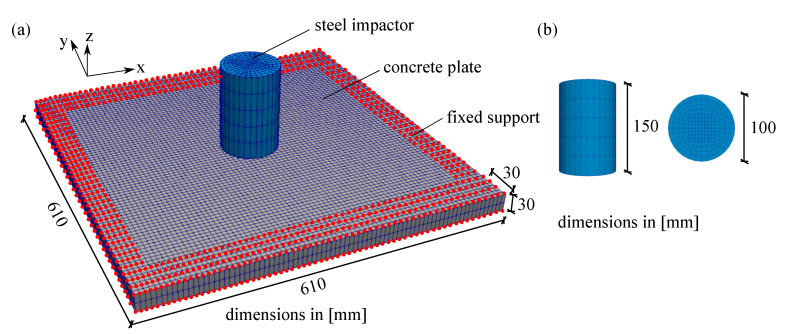
Finite element mesh of thin plate impact test: (**a**) geometry and (**b**) dimensions of the steel impactor.

Second example deals with a square small thin plate subjected to high velocity impact loading based on experiments by [48] with dimension of 610 × 610 × 30 mm. A steel impactor with a diameter of 100 mm and a height of 100 m installed in a steel frame hits the concrete plate by an initial velocity of 12,300 mm/s. As boundary conditions, fixed supports of 30 mm wide denoted by red dots are applied on each side of the plate specimen. Figure 21a displays the whole geometry of the impact test in finite element mesh discretisation, whereas Figure 21b provides the dimension of the steel impactor used in the present work. Due to the symmetry, only a quarter of the geometry is simulated with 5463 nodes and 3440 elements. Moreover, all material properties of this impact test are shown in Table 3.

**Figure 22:**
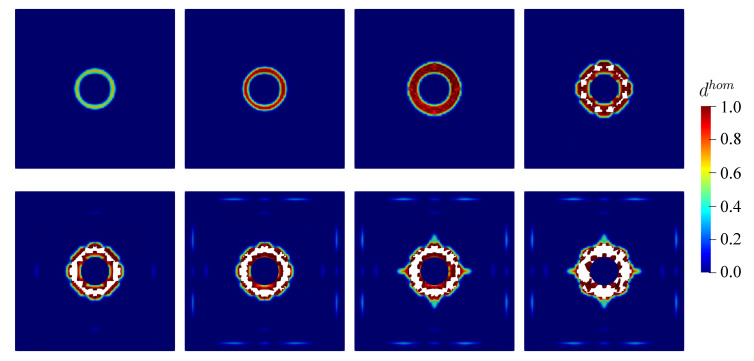
Top view of damage evolution for thin plate impact test at chosen time step.

**Figure 23:**
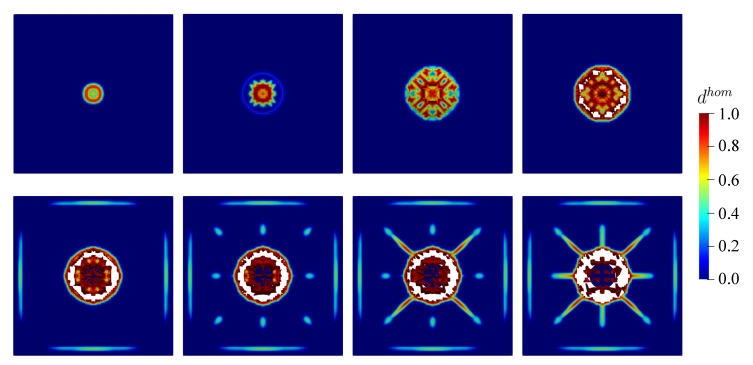
Bottom view of damage evolution for thin plate impact test at chosen time step.

Similar to the previous example, the damage value tolerance $d^{val}$ is considered to be $d^{hom}=0.99$ obtained from the material level. As discussed previously, the closer $d^{val}$ is approaching 1.0, spurious damage in numerical simulations can be avoided. Furthermore, the damage evolution obtained by the proposed finite strain model is displayed in Figure 22 and Figure 23 for the top and bottom side of the concrete plate, respectively. Damage initiation starts at the middle of the plate after the impactor hits the plate surface. Hereafter, damage evolves continuously, in particular surrounding the steel impactor. Then, it gets wider following the impactor velocity. This condition causes the destruction of elements at the mid-surface as shown in Figure 22, while the impactor starts to penetrate into the plate. Subsequently, as one can see in Figure 23, cracks propagate diagonally towards the four corners of the plate emerged at the bottom side. Moreover, vertical and horizontal cracks are then observed.

**Figure 24:**
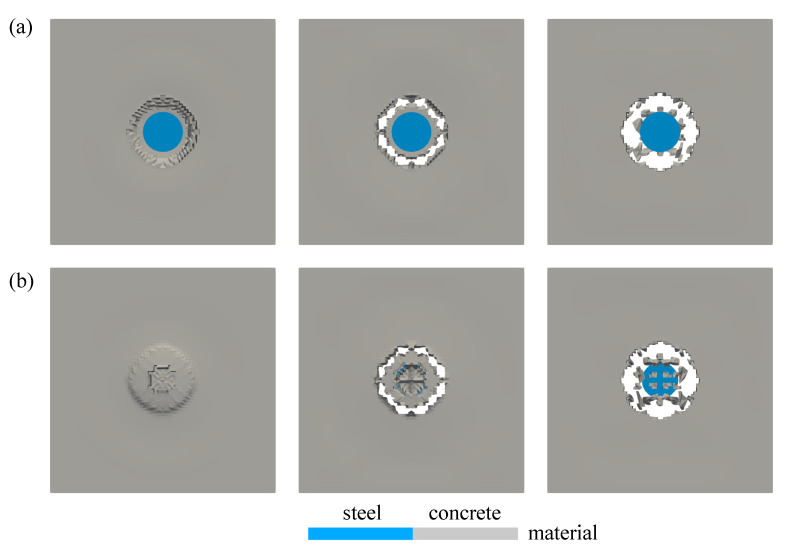
Crack growth visualisation of thin plate impact test: (**a**) top and (**b**) bottom view.

In a similar manner to the preceding example, the crack growth visualisation for this impact simulation is then provided in Figure 24a,b shown from the top and bottom side of the concrete plate, respectively. For the sake of a clearer and more realistic view, Figure 24 displays only the material colour of the steel and concrete. From the figure, additional crack propagations as seen in Figure 23 are not represented in Figure 24b since the damage value $d^{hom}$ of the diagonal cracks as well as the vertical and horizontal cracks have not achieved 0.99 yet.

**Figure 25:**
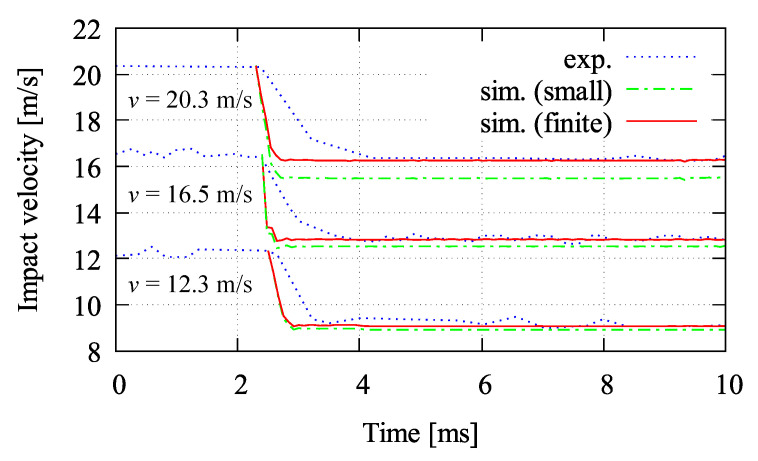
Velocity-time curves reported in [48] compared to small and finite strain simulations for three different velocities of 12.3 m/s, 16.5 m/s, and 20.3 m/s.

To investigate the difference between small and finite strain simulations, the impact test is performed using three different velocities, i.e., 12.3 m/s, 16.5 m/s, and 20.3 m/s. First investigation is the velocity-time relation for both models compared to the experimental data in [48], see Figure 25. At the lower velocity, v=12.3 m/s, coinciding curves of small and finite strain results are observed. Meanwhile, both curves differs slightly at higher velocity than before, with v=16.5 m/s. Furthermore, at v=20.3 m/s, the deviation of both models gets larger with the difference in velocity of approximately 1 m/s. By performing all simulations with the three different velocities, the finite strain microplane model gives better agreement compared to the experimental investigations than the small strain formulation. Here, one can deduce that the higher the impact velocity, the larger the deviation of residual velocity between the small and finite strain simulation. Again, finite strain condition may occur in the impact test simulation at higher velocities, so that the deviation is observed using the small strain simulation.

**Figure 26:**
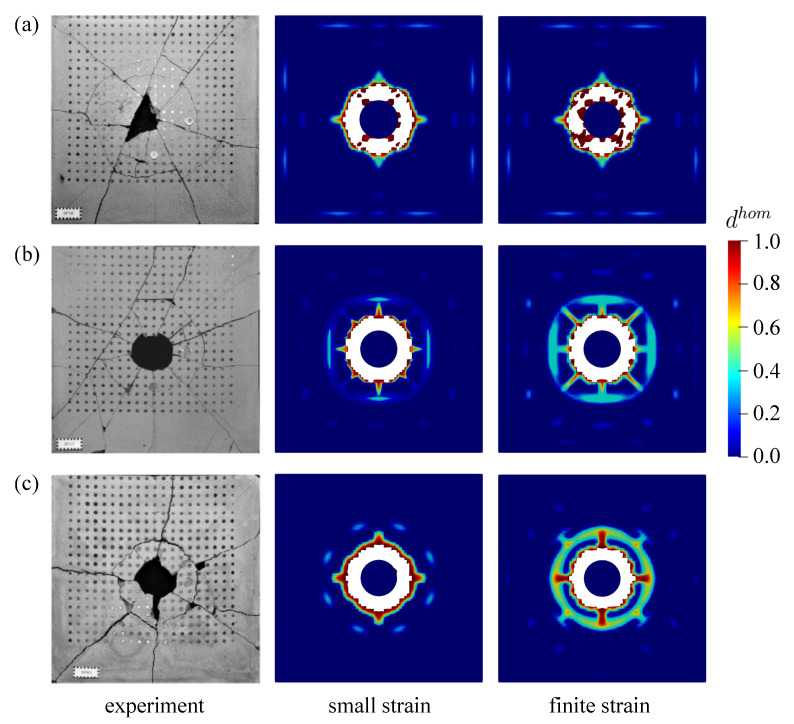
Top view of thin plate impact test for experiments in [48] comparing small and finite strain simulations at three different velocities: (**a**) 12.3 m/s, (**b**) 16.5 m/s, and (**c**) 20.3 m/s.

**Figure 27:**
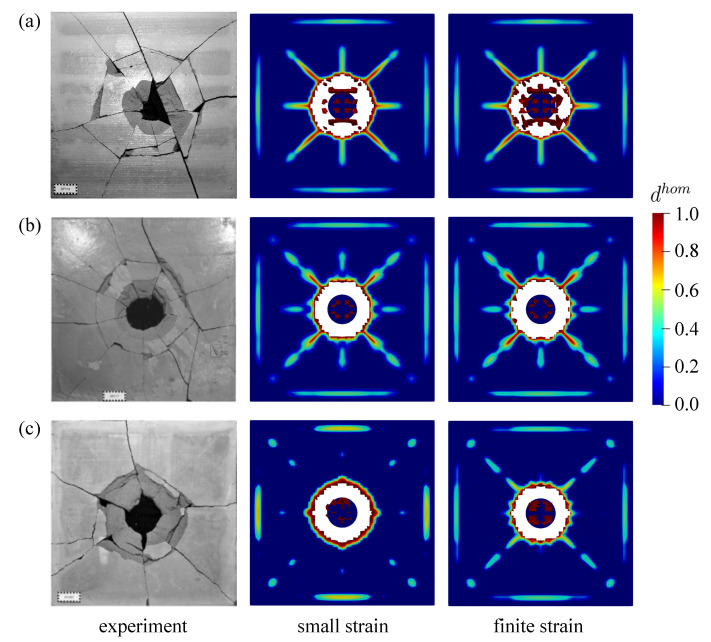
Bottom view of thin plate impact test for experiments in [48] comparing small and finite strain simulations at three different velocities: (**a**) 12.3 m/s, (**b**) 16.5 m/s, and (**c**) 20.3 m/s.

**Figure 28:**
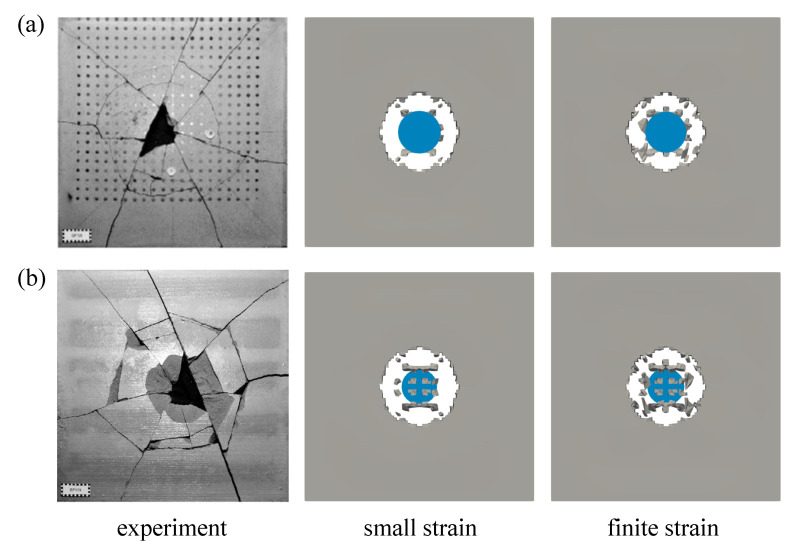
Damaged plate due to impact tests reported in [48] compared to small and finite strain simulations at the velocity of 12.3 m/s: (**a**) top view and (**b**) bottom view.

Second investigation deals with the crack pattern for the small and the finite strain model compared to the experimental data as reported in [48], see Figure 26 and Figure 27 for the top and bottom side view of the plate, respectively. As can be seen in Figure 26a and Figure 27a, almost similar results between the small and finite strain simulations are identified at the lower impact velocity of 12.3 m/s. Meanwhile, a slight difference of both models appears at the impact velocity of 16.5 m/s at the top surface as in Figure 26b, while the damage pattern at the bottom side shown in Figure 27b is almost identical for both models. Moreover, at the higher impact velocity of 20.3 m/s as shown in Figure 26c and Figure 27c, damage patterns obtained by the small strain simulation are focused only in the middle part of the plate. No other crack propagation at the top side is observed unlike the finite strain result. Furthermore, by comparing the simulation results numerically to the existing experimental investigations, one can observe that the finite strain microplane model yields a better accuracy in predicting the damage pattern of the present high velocity impact test than the small strain formulation. Apparently, as can be seen in Figure 28, the difference of crack prediction for both models compared to the experiments is caused by the heterogeneous nature of the concrete material due to its composition as well as aggregate size. In addition, imperfection of the experimental set-up can possibly happen during the impact test in the reality, whereas the numerical simulations are always carried out by considering the model testing in a perfect condition.

Throughout the simulations and result analyses of the small thin concrete plate at the three different velocities, both small and finite strain microplane models are used to perform the simulation. As one can see from the aforementioned investigations, the small strain approach provides good results and gives almost identical responses at the impact velocity of 12.3 m/s compared to the proposed finites strain formulation. Hence, both models are able to simulate this condition. However, each formulation yields different results during the impact test at higher velocities, such as 16.5 m/s and 20.3 m/s. The observation in terms of velocity and crack pattern simulated by the finite strain model gives a better approximation compared to the experimental data than the small strain formulation. In this regard, a finite strain case probably occurs so that the small strain microplane model cannot suffice to demonstrate the accurate response.

## 7. Conclusions and Outlook

Formulating a robust material model for concrete using the well-known microplane approach extended to the finite strain framework as a main objective of the contribution at hand is achieved. The proposed model can be used to analyse concrete structures numerically at various loading states particularly for dynamic cases within implicit finite element codes. Constitutive models are developed in the present work involving a combination of both damage and plasticity. The newly proposed model combines the plastic-damage microplane approach at finite strains due to the fact that large deformations occur in concrete at high velocity impacts. The conjugate pair of the Green-Lagrange strain tensor and the second Piola-Kirchhoff stress tensor is used to extend the small strain microplane model to the finite strain regime. A smooth three-surface Drucker–Prager yield function is introduced and supplemented by rate dependency to deliver strain rate effects at dynamic loading. The proposed model is then used to simulate impact tests with high velocities on concrete plates after being implemented together with a contact mechanism and an adaptive element erosion during penetration.

A penetration model is required in such impact tests due to the fact that an impactor may penetrate into concrete structures at high velocity impact. Damage values obtained by the proposed material model are used as failure criterion to eliminate destroyed elements of concrete during the impactor penetration. To simulate this phenomenon, the adaptive element erosion is also equipped with a general contact mechanism. The accuracy and capability of the new framework are then evaluated by comparing with the experimental data. As a result, the proposed microplane model at finite strains gives better agreement to the experimental investigations than the small strain version since finite strain situation may occur in the present impact test simulations.

The research work at hand is able to represent concrete responses in a good approximation and reliable results compared to existing experimental investigations. However, some improvements can be pursued to obtain a more sophisticated tool for modelling concrete structures in larger examples and different types of loading. For instance, the adjustment of model parameters in an advanced manner is useful to facilitate numerical simulations of other cases. Herein, the plastic parameters can be simplified by identifying the uniaxial compressive strength of concrete. Nonetheless, determination of damage and nonlocal parameters in each numerical example are not simple. It probably needs an optimisation algorithm in order to find accurate values, yet indeed, computational costs may increase accordingly.

## Figures and Tables

**Table 1:** Model parameters for four-point bending test.

Parameter		Concrete
*E*	[MPa]	38,000
ν	[-]	0.2
$f_{uc}$	[MPa]	20
$R_{t}$	[-]	1
*D*	[MPa]	$7\times 10^{4}$
$S_{V}^{C}$	[MPa]	−40
*R*	[-]	2
$\gamma_{t0}$	[-]	0
$\gamma_{c0}$	[-]	$2\times 10^{-5}$
$\beta_{t}$	[-]	$5.5\times 10^{3}$
$\beta_{c}$	[-]	$3\times 10^{3}$
*c*	[${\mathrm{mm}}^{2}$]	20
*m*	[-]	2.5

**Table 2:** Material properties for the thick plate impact test.

Parameter		Concrete Plate	Steel Impactor
*E*	[MPa]	40,000	210,000
ν	[-]	0.2	0.3
ρ	[$\mathrm{kg}/m^{3}$]	2400	7850
$f_{uc}$	[MPa]	70	-
$R_{t}$	[-]	1	-
*D*	[MPa]	$7\times 10^{4}$	-
$S_{V}^{C}$	[MPa]	−60	-
*R*	[-]	2	-
$\gamma_{t0}$	[-]	0	-
$\gamma_{c0}$	[-]	$8\times 10^{-5}$	-
$\beta_{t}$	[-]	$5\times 10^{3}$	-
$\beta_{c}$	[-]	$2\times 10^{3}$	-
*c*	[${\mathrm{mm}}^{2}$]	50	-
*m*	[-]	2.5	-
$\eta_{vp}$	[s/MPa]	1	-

**Table 3:** Material properties for the thin plate impact test.

Parameter		Concrete Plate	Steel Impactor
*E*	[MPa]	33,100	210,000
ν	[-]	0.2	0.3
ρ	[$\mathrm{kg}/m^{3}$]	2150	8000
$f_{uc}$	[MPa]	95	-
$R_{t}$	[-]	1	-
*D*	[MPa]	$7\times 10^{4}$	-
$S_{V}^{C}$	[MPa]	−75	-
*R*	[-]	2	-
$\gamma_{t0}$	[-]	0	-
$\gamma_{c0}$	[-]	$8\times 10^{-5}$	-
$\beta_{t}$	[-]	$5\times 10^{3}$	-
$\beta_{c}$	[-]	$2\times 10^{3}$	-
*c*	[${\mathrm{mm}}^{2}$]	20	-
*m*	[-]	2.5	-
$\eta_{vp}$	[s/MPa]	1	-

## Appendix A. Algorithmic Tangent

Algorithmic tangent moduli for the proposed model contain both elastoplastic and damage tangent terms. Once the elastoplastic tangent is obtained, the total tangent is then taken into account by considering damage.

### Appendix A.1. Effective Elastoplastic Tangent

The differentiation of the yield function is required to compute the effective tangent terms

(A1) $$\delta F^{mic}=n_{V}\delta S_{V}^{e}+n_{D}\cdot \delta S_{D}^{e}+n_{\kappa }\delta \kappa =0,$$

with

(A2) $$n_{V}=\frac{\partial F^{mic}}{\partial S_{V}^{e}},\;n_{D}=\frac{\partial F^{mic}}{\partial S_{D}^{e}},\;n_{\kappa }=\frac{\partial F^{mic}}{\partial \kappa }.$$

Next, the volumetric and deviatoric stresses are differentiated as

(A3) $$\delta S_{V}^{e}=K^{mic}\left(\delta E_{V}-\delta \lambda m_{V}-\Delta \lambda \frac{\partial m_{V}}{\partial S_{V}^{e}}\delta S_{V}^{e}-\Delta \lambda \frac{\partial m_{V}}{\partial \kappa }\delta \kappa \right),$$

(A4) $$\delta S_{D}^{e}=2G^{mic}\left(\delta E_{D}-\delta \lambda m_{D}-\Delta \lambda \frac{\partial m_{D}}{\partial S_{D}^{e}}\cdot \delta S_{D}^{e}\right).$$

Knowing Equation (21), Equation (A3) becomes

(A5) $$\delta S_{V}^{e}=K^{mic}\left(\delta E_{V}-\delta \lambda m_{V}-\Delta \lambda \frac{\partial m_{V}}{\partial S_{V}^{e}}\delta S_{V}^{e}-\Delta \lambda \frac{\partial m_{V}}{\partial \kappa }\delta \lambda \right).$$

Then, one can rearrange Equations (A4) and (A5) as

(A6) $$\delta S_{V}^{e}\left(\frac{1}{K^{mic}}+\Delta \lambda \frac{\partial m_{V}}{\partial S_{V}^{e}}\right)=\delta E_{V}-\delta \lambda \left(m_{V}+\Delta \lambda \frac{\partial m_{V}}{\partial \kappa }\right),$$

(A7) $$\left(\frac{1}{2G^{mic}}+\Delta \lambda \frac{\partial m_{D}}{\partial S_{D}^{e}}\right)\cdot \delta S_{D}^{e}=\delta E_{D}-\delta \lambda m_{D}.$$

Furthermore, Equations (A6) and (A7) can be written as

(A8) $$\delta S_{V}^{e}=h_{1}\delta E_{V}-h_{1}h_{2}\delta \lambda ,$$

(A9) $$S_{D}^{e}=H_{3}\cdot \delta E_{D}-H_{3}\cdot m_{D}\delta \lambda ,$$

where $h_{1}$, $h_{2}$, and $H_{3}$ are, respectively,

(A10) $$h_{1}={\left(\frac{1}{K^{mic}}+\Delta \lambda \frac{\partial m_{V}}{\partial S_{V}^{e}}\right)}^{-1},$$

(A11) $$h_{2}=m_{V}+\Delta \lambda \frac{\partial m_{V}}{\partial \kappa },$$

(A12) $$H_{3}={\left(\frac{1}{2G^{mic}}+\Delta \lambda \frac{\partial m_{D}}{\partial S_{D}^{e}}\right)}^{-1}.$$

Now, Equations (A8) and (A9) are substituted into Equation (A1) as follows

(A13) $$\delta \lambda =\frac{n_{V}h_{1}\delta E_{V}+n_{D}\cdot H_{3}\cdot \delta E_{D}}{\left(n_{v}h_{1}h_{2}+n_{D}\cdot H_{3}\cdot m_{D}-\eta_{\kappa }\right)},$$

or one can write in a convenient way

(A14) $$\delta \lambda =h_{1}h_{4}n_{V}\delta E_{V}+h_{4}n_{D}\cdot H_{3}\cdot \delta E_{D},$$

where $h_{4}$ is

(A15) $$h_{4}={\left(n_{v}h_{1}h_{2}+n_{D}\cdot H_{3}\cdot m_{D}-\eta_{\kappa }\right)}^{-1}.$$

Then, Equation (A14) can be substituted into Equations (A8) and (A9)

(A16) $$\delta S_{V}^{e}=h_{1}\delta E_{V}-h_{1}h_{2}\left(h_{1}h_{4}n_{V}\delta E_{V}+h_{4}n_{D}\cdot H_{3}\cdot \delta E_{D}\right),$$

(A17) $$S_{D}^{e}=H_{3}\cdot \delta E_{D}-H_{3}\cdot m_{D}\left(h_{1}h_{4}n_{V}\delta E_{V}+h_{4}n_{D}\cdot H_{3}\cdot \delta E_{D}\right).$$

Subsequently, multiplying Equations (A16) and (A17) by V and ${\mathrm{Dev}}^{T}$, respectively, one obtains

(A18) $$V\delta S_{V}^{e}=h_{1}V\delta E_{V}-h_{1}h_{1}h_{2}h_{4}n_{V}V\delta E_{V}-h_{1}h_{2}h_{4}V⊗n_{D}\cdot H_{3}\cdot \delta E_{D},$$

(A19) $$\begin{array}{cc} {\mathrm{Dev}}^{T}\cdot \delta S_{D}^{e} & ={\mathrm{Dev}}^{T}\cdot H_{3}\cdot \delta E_{D}-h_{1}h_{4}n_{V}{\mathrm{Dev}}^{T}\cdot H_{3}\cdot m_{D}\delta E_{V}\\ \; & -h_{4}{\mathrm{Dev}}^{T}\cdot H_{3}\cdot m_{D}⊗n_{D}\cdot H_{3}\cdot \delta E_{D}. \end{array}$$

The differentiation of the effective second Piola-Kirchhoff stress tensor for one microplane is obtained by summing Equations (A18) and (A19) as

(A20) $$\begin{array}{cc} \delta S_{e}^{mic} & =V\delta S_{V}^{e}+{\mathrm{Dev}}^{T}\cdot \delta S_{D}^{e}\\ \; & =(\left(1-h_{1}h_{2}h_{4}n_{V}\right)h_{1}V⊗V-h_{1}h_{4}n_{V}{\mathrm{Dev}}^{T}\cdot H_{3}\cdot m_{D}⊗V\\ \; & -h_{1}h_{2}h_{4}V⊗n_{D}\cdot H_{3}\cdot \mathrm{Dev}+{\mathrm{Dev}}^{T}\cdot H_{3}\cdot \mathrm{Dev}\\ \; & -h_{4}{\mathrm{Dev}}^{T}\cdot H_{3}\cdot m_{D}⊗n_{D}\cdot H_{3}\cdot \mathrm{Dev}):\delta E. \end{array}$$

Now, $\delta S_{e}^{mic}$ is used to obtain the total tangent incorporated with damage.

### Appendix A.2. Total Tangent

From Equation (11), one can write

(A21) $$S=\frac{3}{4\pi }\int_{\Omega }\left(1-d^{mic}\right)S_{e}^{mic}\;d\Omega ,$$

where $S_{e}^{mic}$ originates from

(A22) $$S_{e}^{mic}=K^{mic}V\left(E_{V}-E_{V}^{p}\right)+2G^{mic}{\mathrm{Dev}}^{T}\cdot \left(E_{D}-E_{D}^{p}\right).$$

Then, Equation (A21) is differentiated as

(A23) $$\delta S=\frac{3}{4\pi }\int_{\Omega }\left(1-d^{mic}\right)\delta S_{e}^{mic}\;d\Omega -\frac{3}{4\pi }\int_{\Omega }S_{e}^{mic}\delta d^{mic}\;d\Omega ,$$

where $\delta S_{e}^{mic}$ is obtained from Equation (A20), while $\delta d^{mic}$ is

(A24) $$\delta d^{mic}=\frac{\partial d^{mic}}{\partial E}:\delta E+\frac{\partial d^{mic}}{\partial {\bar{\eta }}_{mc}}\delta {\bar{\eta }}_{mc}+\frac{\partial d^{mic}}{\partial {\bar{\eta }}_{mt}}\delta {\bar{\eta }}_{mt},$$

which is differentiated with respect to both local and nonlocal quantities in terms of compression and tensions part, so that

(A25) $$\frac{\partial d^{mic}}{\partial E}=\left(1-r_{w}d_{t}^{mic}\right)\frac{\partial d_{c}^{mic}}{\partial \eta^{mic}}\frac{\partial \eta^{mic}}{\partial E}+\frac{\partial d^{mic}}{\partial r_{w}}\frac{\partial r_{w}}{\partial E}+\left(1-d_{c}^{mic}\right)r_{w}\frac{\partial d_{t}^{mic}}{\partial \eta^{mic}}\frac{\partial \eta^{mic}}{\partial E},$$

(A26) $$\frac{\partial d_{c}^{mic}}{\partial \eta^{mic}}=\frac{\partial d_{c}^{mic}}{\partial \gamma_{c}^{mic}}\frac{\partial \gamma_{c}^{mic}}{\partial {\hat{\eta }}_{c}^{mic}}\left(1-m\right)\left(1-r_{w}\right),$$

(A27) $$\frac{\partial d_{t}^{mic}}{\partial \eta^{mic}}=\frac{\partial d_{t}^{mic}}{\partial \gamma_{t}^{mic}}\frac{\partial \gamma_{t}^{mic}}{\partial {\hat{\eta }}_{t}^{mic}}\left(1-m\right)r_{w},$$

with the required derivatives as follows

(A28) $$\frac{\partial d_{c}^{mic}}{\partial \gamma_{c}^{mic}}=\beta_{c}\;\exp\left(-\beta_{c}\gamma_{c}^{mic}\right),$$

(A29) $$\frac{\partial d_{t}^{mic}}{\partial \gamma_{t}^{mic}}=\beta_{t}\;\exp\left(-\beta_{t}\gamma_{t}^{mic}\right),$$

(A30) $$\frac{\partial \gamma_{c}^{mic}}{\partial {\hat{\eta }}_{c}^{mic}}=\left\{\begin{array}{cc} 1 & \;\mathrm{for}\;{\hat{\eta }}_{c}^{mic}>\gamma_{c0}\\ 0 & \;\mathrm{for}\;{\hat{\eta }}_{c}^{mic}\leq \gamma_{c0} \end{array}\right.,$$

(A31) $$\frac{\partial \gamma_{t}^{mic}}{\partial {\hat{\eta }}_{t}^{mic}}=\left\{\begin{array}{cc} 1 & \;\mathrm{for}\;{\hat{\eta }}_{t}^{mic}>\gamma_{t0}\\ 0 & \;\mathrm{for}\;{\hat{\eta }}_{t}^{mic}\leq \gamma_{t0} \end{array}\right.,$$

(A32) $$\frac{\partial \eta^{mic}}{\partial E}=\left\{\begin{array}{cc} \frac{\partial \Delta \lambda }{\partial E}m_{V}+\Delta \lambda \frac{\partial m_{V}}{\partial E} & \;\mathrm{for}\;{\dot{E}}_{V}^{p}>0\\ 0 & \;\mathrm{for}\;{\dot{E}}_{V}^{p}\leq 0 \end{array}\right..$$

Meanwhile, one can obtain the derivatives of Δλ and $m_{V}$ with respect to the strain tensor E as

(A33) $$\frac{\partial \Delta \lambda }{\partial E}=h_{4}\left(h_{1}n_{V}V+n_{D}\cdot H_{3}\cdot \mathrm{Dev}\right),$$

(A34) $$\frac{\partial m_{V}}{\partial E}=\frac{\partial m_{V}}{\partial S_{V}^{e}}\frac{\partial S_{V}^{e}}{\partial E}+\frac{\partial m_{V}}{\partial \Delta \lambda }\frac{\partial \Delta \lambda }{\partial E},$$

(A35) $$\frac{\partial S_{V}^{e}}{\partial E}=h_{1}\left(1-h_{1}h_{2}h_{4}n_{V}\right)V-h_{1}h_{2}h_{4}n_{D}\cdot H_{3}\cdot \mathrm{Dev}.$$

Next, one can derive damage with respect to the split factor $r_{w}$ as

(A36) $$\frac{\partial d^{mic}}{\partial r_{w}}=\left(1-r_{w}d_{t}^{mic}\right)\frac{\partial d_{c}^{mic}}{\partial r_{w}}+\left(1-d_{c}^{mic}\right)\frac{\partial d_{t}^{mic}}{\partial r_{w}}+\left(1-d_{c}^{mic}\right)d_{t}^{mic},$$

(A37) $$\frac{\partial d_{c}^{mic}}{\partial r_{w}}=-\frac{\partial d_{c}^{mic}}{\partial \gamma_{c}^{mic}}\frac{\partial \gamma_{c}^{mic}}{\partial {\hat{\eta }}_{c}^{mic}}\left(1-m\right)\Delta \lambda m_{V},$$

(A38) $$\frac{\partial d_{t}^{mic}}{\partial r_{w}}=\frac{\partial d_{t}^{mic}}{\partial \gamma_{t}^{mic}}\frac{\partial \gamma_{t}^{mic}}{\partial {\hat{\eta }}_{t}^{mic}}r_{w}\left(1-m\right)\Delta \lambda m_{V},$$

(A39) $$\frac{\partial r_{w}}{\partial E}=\sum_{I=1}^{3}\left(\frac{\partial r_{w}}{\partial E^{I}}\frac{\partial E^{I}}{\partial E}\right),$$

(A40) $$\frac{\partial r_{w}}{\partial E^{I}}=\frac{\left(\sum_{I=1}^{3}\left|E^{I}\right|\right)\frac{\partial \langle E^{I}\rangle }{\partial E^{I}}-\left(\sum_{I=1}^{3}\langle E^{I}\rangle \right)\frac{\partial |E^{I}|}{\partial E^{I}}}{{\left(\sum_{I=1}^{3}\left|E^{I}\right|\right)}^{2}},$$

(A41) $$\frac{\partial E^{I}}{\partial E}=u_{I}⊗u_{I},$$

where $u_{I}$ denotes the eigenvector that corresponds to the eigenvalue $E_{I}$.

Finally, the macroscopic total tangent terms as well as the stress derivatives with respect to the nonlocal quantities in terms of compression and tension parts are

(A42) $$\frac{\partial S}{\partial E}=\frac{3}{4\pi }\int_{\Omega }\left(1-d^{mic}\right)\delta S_{e}^{mic}\;d\Omega -\frac{3}{4\pi }\int_{\Omega }S_{e}^{mic}⊗\frac{\partial d^{mic}}{E}\;d\Omega ,$$

(A43) $$\frac{\partial S}{\partial {\bar{\eta }}_{mc}}=-\frac{3}{4\pi }\int_{\Omega }S_{e}^{mic}\left(1-r_{w}d_{t}^{mic}\right)\frac{\partial d_{c}^{mic}}{\partial {\bar{\eta }}_{mc}}\;d\Omega ,$$

(A44) $$\frac{\partial S}{\partial {\bar{\eta }}_{mt}}=-\frac{3}{4\pi }\int_{\Omega }S_{e}^{mic}\left(1-d_{c}^{mic}\right)\frac{\partial d_{t}^{mic}}{\partial {\bar{\eta }}_{mt}}\;d\Omega ,$$

where

(A45) $$\frac{\partial d_{c}^{mic}}{\partial {\bar{\eta }}_{mc}}=\frac{\partial d_{c}^{mic}}{\partial \gamma_{c}^{mic}}\frac{\partial \gamma_{c}^{mic}}{\partial {\hat{\eta }}_{c}^{mic}}\left(1-m\right),$$

(A46) $$\frac{\partial d_{t}^{mic}}{\partial {\bar{\eta }}_{mt}}=\frac{\partial d_{t}^{mic}}{\partial \gamma_{t}^{mic}}\frac{\partial \gamma_{t}^{mic}}{\partial {\hat{\eta }}_{t}^{mic}}m,$$

and the derivatives of the local variable in compression and tension with respect to the Green-Lagrange strain tensor are

(A47) $$\frac{\partial \eta_{mc}}{\partial E}=\frac{1}{4\pi }\int_{\Omega }\left(\left(1-r_{w}\right)\frac{\partial \eta^{mic}}{\partial E}-\frac{\partial r_{w}}{\partial E}\Delta \lambda m_{V}\right)d\Omega ,$$

(A48) $$\frac{\partial \eta_{mt}}{\partial E}=\frac{1}{4\pi }\int_{\Omega }\left(r_{w}\frac{\partial \eta^{mic}}{\partial E}+\frac{\partial r_{w}}{\partial E}\Delta \lambda m_{V}\right)d\Omega .$$

All required derivatives for calculating the tangent stiffness in the element level as in Equations (63)–(66) should be transformed to the current configuration using the push-forward operation.

## References

[1] Cicekli U., Voyiadjis G.Z., Al-Rub R.K.A. (2007). A plasticity and anisotropic damage model for plain concrete. Int. J. Plast..

[2] Grassl P., Jirásek M. (2006). Damage-plastic model for concrete failure. Int. J. Solids Struct..

[3] Grassl P., Xenos D., Nyström U., Rempling R., Gylltoft K. (2013). CDPM2: A damage-plasticity approach to modelling the failure of concrete. Int. J. Solids Struct..

[4] Jason L., Huerta A., Pijaudier-Cabot G., Ghavamian S. (2006). An elastic plastic damage formulation for concrete: Application to elementary tests and comparison with an isotropic damage model. Comput. Methods Appl. Mech. Eng..

[5] Lee J., Fenves G.L. (1998). Plastic-damage model for cyclic loading of concrete structures. J. Eng. Mech..

[6] Meschke G., Lackner R., Mang H.A. (1998). An anisotropic elastoplastic-damage model for plain concrete. Int. J. Numer. Methods Eng..

[7] Nguyen G.D., Houlsby G.T. (2008). A coupled damage-plasticity model for concrete based on thermodynamic principles: Part I: Model formulation and parameter identification. Int. J. Numer. Anal. Methods Geomech..

[8] Voyiadjis G.Z., Taqieddin Z.N., Kattan P.I. (2008). Anisotropic damage-plasticity model for concrete. Int. J. Plast..

[9] Zhang J., Li J., Ju J.W. (2016). 3D elastoplastic damage model for concrete based on novel decomposition of stress. Int. J. Solids Struct..

[10] Ahad F.R., Enakoutsa K., Solanki K.N., Bammann D.J. (2014). Nonlocal modelling in high-velocity impact failure of 6061-T6 aluminum. Int. J. Plast..

[11] Majorana C.E., Salomoni V.A., Mazzucco G., Khoury G.A. (2010). An approach for modelling concrete spalling in finite strains. Math. Comput. Simul..

[12] Nguyen G.D., Korsunsky A.M., Belnoue J.P.H. (2015). A nonlocal coupled damage-plasticity model for the analysis of ductile failure. Int. J. Plast..

[13] Zhu Q.Z., Shao J.F., Mainguy M. (2010). A micromechanics-based elastoplastic damage model for granular materials at low confining pressure. Int. J. Plast..

[14] Bažant Z.P., Oh B. Microplane model for concrete for fracture analysis of concrete structures. Proceedings of the Symposium on the Interaction of Non-nuclear Munitions with Structures, U.S. Air Force Academy.

[15] Bažant Z.P., Gambarova P.G. (1984). Crack shear in concrete: Crack band microplane model. J. Struct. Eng..

[16] Caner F., Bažant Z.P. (2013). Microplane model M7 for plain concrete. I: Formulation. J. Eng. Mech..

[17] Kuhl E., Ramm E. (2000). Microplane modelling of cohesive frictional materials. Eur. J. Mech. A Solids.

[18] Kuhl E. (2000). Numerische Modelle Für Kohäsive Reibungsmaterialien. Ph.D. Thesis.

[19] Leukart M. (2005). Kombinierte Anisotrope Schädigung und Plastizität bei Kohäsiven Reibungsmaterialien. Ph.D. Thesis.

[20] Peerlings R.H.J., de Borst R., Brekelmans W.A.M., de Vree J.H.P. (1996). Gradient enhanced damage for quasi-brittle materials. Int. J. Numer. Methods Eng..

[21] Peerlings R.H.J., Geers M.G.D., de Borst R., Brekelmans W.A.M. (2001). A critical comparison of nonlocal and gradient-enhanced softening continua. Int. J. Solids Struct..

[22] Zreid I., Kaliske M. (2014). Regularization of microplane damage models using an implicit gradient enhancement. Int. J. Solids Struct..

[23] Zreid I., Kaliske M. (2016). An implicit gradient formulation for microplane Drucker-Prager plasticity. Int. J. Plast..

[24] Zreid I., Kaliske M. (2018). A gradient-enhanced plasticity-damage microplane model for concrete. Comput. Mech..

[25] Bažant Z.P., Kim J.J.H., Brocca M. (1999). Finite strain tube-squash test of concrete at high pressures and shear angles up to 70 degrees. ACI Mater. J..

[26] Indriyantho B.R., Zreid I., Kaliske M. (2019). Finite strain extension of a gradient enhanced microplane damage model for concrete at static and dynamic loading. Eng. Fract. Mech..

[27] Indriyantho B.R., Kaliske M. (2019). A finite strain microplane-plasticity model for concrete. Proceedings of the 8th GACM Colloquium on Computational Mechanics for Young Scientists from Academia and Industry, German Association for Computational Mechanics (GACM).

[28] Indriyantho B.R., Zreid I., Kaliske M. (2020). A nonlocal softening plasticity based on microplane theory for concrete at finite strains. Comput. Struct..

[29] Bažant Z.P., Adley M.D., Carol I., Jirásek M., Akers S.A., Rohani B., Cargile J.D., Caner F.C. (2000). Large-strain generalization of microplane model for concrete and application. J. Eng. Mech..

[30] Huang F., Wu H., Jin Q., Zhang Q. (2005). A numerical simulation on the perforation of concrete targets. Int. J. Impact Eng..

[31] Bažant Z.P. (1996). Finite strain generalization of small-strain constitutive relations for any finite strain tensor and additive volumetic-deviatoric split. Int. J. Solids Struct..

[32] Lee E.H. (1969). Elastic-plastic deformation at finite strains. J. Appl. Mech..

[33] Dolarevic S., Ibrahimbegovic A. (2007). A modified three-surface elasto-plastic cap model and its numerical implementation. Comput. Struct..

[34] Schwer L.E., Murray Y.D. (1994). A three-invariant smooth cap model with mixed hardening. Int. J. Numer. Anal. Methods Geomech..

[35] Jiang H., Zhao J. (2015). Calibration of the continuous surface cap model for concrete. Finite Elem. Anal. Des..

[36] Wang W.M., Sluys L.J., de Borst R. (1997). Viscoplasticity for instabilities due to strain softening and strain-rate softening. Int. J. Numer. Methods Eng..

[37] Fuchs A., Kaliske M., Pijaudier-Cabot G., Grassl P., La Borderie C. (2019). A gradient enhanced viscoplasticity-damage microplane model for concrete at static and transient loading. Proceedings of the 10th International Conference on Fracture Mechanics of Concrete and Concrete Structures.

[38] Qinami A., Pandolfi A., Kaliske M. (2020). Variational eigenerosion for rate-dependent plasticity in concrete modelling at small strain. Int. J. Numer. Methods Eng..

[39] Grassl P., Jirásek M. (2006). Plastic model with non-local damage applied to concrete. Int. J. Numer. Anal. Methods Geomech..

[40] Poh L.H., Swaddiwudhipong S. (2009). Over-nonlocal gradient enhanced plastic-damage model for concrete. Int. J. Solids Struct..

[41] Ožbolt J., Bošnjak J., Sola E. (2013). Dynamic fracture of concrete compact tension specimen: Experimental and numerical study. Int. J. Solids Struct..

[42] Bažant Z.P., Pijaudier-Cabot G. (1989). Measurement of characteristic length of nonlocal continuum. J. Eng. Mech..

[43] Le Bellégo C., Dubé J.F., Pijaudier-Cabot G., Gérard B. (2003). Calibration of nonlocal damage model from size effect tests. Eur. J. Mech. A Solids.

[44] Xenos D., Grégoire D., Morel S., Grassl P. (2015). Calibration of nonlocal models for tensile fracture in quasi-brittle heterogeneous materials. J. Mech. Phys. Solids.

[45] Di Luzio G. (2007). A symmetric over-nonlocal microplane model M4 for fracture in concrete. Int. J. Solids Struct..

[46] Hordijk D.A. (1991). Local Approach to Fatigue of Concrete. Ph.D. Thesis.

[47] Qinami A., Zreid I., Fleischhauer R., Kaliske M. (2016). Modeling of impact on concrete plates by use of the microplane approach. Int. J. Non-Linear Mech..

[48] Hering M., Kühn T., Curbach M. (2020). Small-scale plate tests with fine concrete in experiment and first simplified simulation. Struct. Concr..

